# Cross-reactive carbohydrate determinant-specific IgE obscures true atopy and exhibits α-1,3-fucose epitope-specific inverse associations with asthma

**DOI:** 10.1111/all.14469

**Published:** 2020-07-08

**Authors:** Gyaviira Nkurunungi, Harriet Mpairwe, Serge A. Versteeg, Angela van Diepen, Jacent Nassuuna, Joyce Kabagenyi, Irene Nambuya, Richard E. Sanya, Margaret Nampijja, Sonia Serna, Niels-Christian Reichardt, Cornelis H. Hokke, Emily L. Webb, Ronald van Ree, Maria Yazdanbakhsh, Alison M. Elliott

**Affiliations:** 1 Immunomodulation and Vaccines Programme, Medical Research Council/Uganda Virus Research Institute and London School of Hygiene and Tropical Medicine (MRC/UVRI and LSHTM) Uganda Research Unit, Entebbe, Uganda; 2 Department of Clinical Research, London School of Hygiene and Tropical Medicine, London, UK; 3 Departments of Experimental Immunology and of Otorhinolaryngology, Amsterdam University Medical Centers (AMC), Amsterdam, The Netherlands; 4 Department of Parasitology, Leiden University Medical Center, Leiden, The Netherlands; 5 College of Health Sciences, Makerere University, Kampala, Uganda; 6 Glycotechnology Laboratory, Centro de Investigación Cooperativa en Biomateriales (CIC biomaGUNE), San Sebastián, Spain; 7 Centro de Investigación Biomédica en Red en Bioingeniería, Biomateriales y Nanomedicina (CIBER-BBN), San Sebastián, Spain; 8 Department of Infectious Disease Epidemiology, London School of Hygiene and Tropical Medicine, MRC Tropical Epidemiology Group, London, UK

**Keywords:** asthma, cross-reactive carbohydrate determinant, *Schistosoma mansoni*, α-1,3-fucose, β-1,2-xylose

## Abstract

**Background:**

In high-income, temperate countries, IgE to allergen extracts is a risk factor for, and mediator of, allergy-related diseases (ARDs). In the tropics, positive IgE tests are also prevalent, but rarely associated with ARD. Instead, IgE responses to ubiquitous cross-reactive carbohydrate determinants (CCDs) on plant, insect and parasite glycoproteins, rather than to established major allergens, are dominant. Because anti-CCD IgE has limited clinical relevance, it may impact ARD phenotyping and assessment of contribution of atopy to ARD.

**Methods:**

Using an allergen extract-based test, a glycan and an allergen (glyco)protein microarray, we mapped IgE fine specificity among Ugandan rural *Schistosoma mansoni* (*Sm*)-endemic communities, proximate urban communities, and importantly in asthmatic and nonasthmatic schoolchildren.

**Results:**

Overall, IgE sensitization to extracts was highly prevalent (43%-73%) but allergen arrays indicated that this was not attributable to established major allergenic components of the extracts (0%-36%); instead, over 40% of all participants recognized CCD-bearing components. Using glycan arrays, we dissected IgE responses to specific glycan moieties and found that reactivity to classical CCD epitopes (core β-1,2-xylose, α-1,3-fucose) was positively associated with sensitization to extracts, rural environment and *Sm* infection, but not with skin reactivity to extracts or sensitization to their major allergenic components. Interestingly, we discovered that reactivity to only a subset of core α-1,3-fucose-carrying N-glycans was inversely associated with asthma.

**Conclusions:**

CCD reactivity is not just an epiphenomenon of parasite exposure hampering specificity of allergy diagnostics; mechanistic studies should investigate whether specific CCD moieties identified here are implicated in the protective effect of certain environmental exposures against asthma.

## Graphical abstract

**Graphical Abstract:**
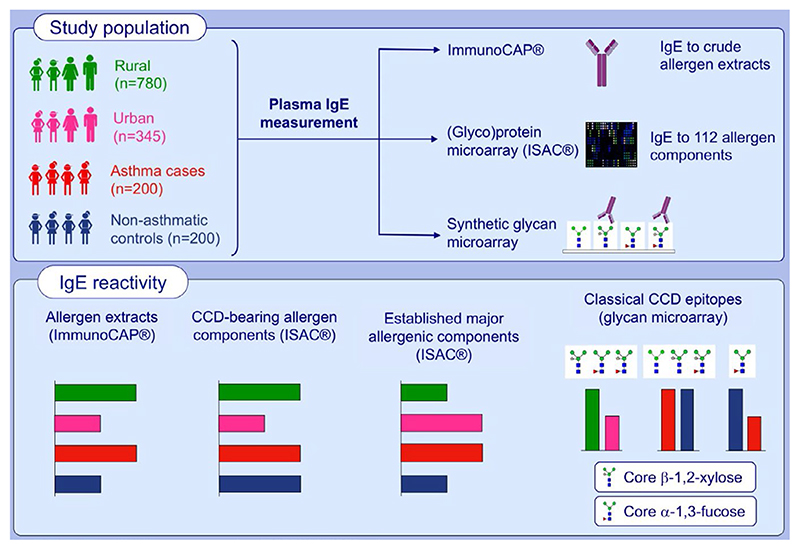
Reactivity to crude allergen extracts and classical CCD epitope (core β-1,2-xylose, α-1,3-fucose)-modified N-glycans is positively associated with the rural, *Schistosoma mansoni-endemic* environment. However, a higher proportion of urban, compared to rural participants, recognise recombinant established major allergenic components. There are inverse associations between reactivity to a subset of core α-1,3-fucosesubstituted N-glycans and asthma, but not for CCD-specific IgE in general.

## 1 Introduction

In high-income countries, IgE to standard allergen extracts is a risk factor for, and mediator of, allergy-related diseases, including asthma, and defines “atopic” allergy-related disease phenotypes.^1–3^ In tropical low-income countries (LICs), allergen extract-specific IgE levels are often elevated, but rarely associated with allergy-related disease.^4^ Host immune responses to common allergen sources exhibit important parallels with responses to parasite, insect and other environmental exposures that have related molecular targets.^5^ For example, the *Onchocerca volvulus* tropomyosin protein is structurally homologous to house dust mite (HDM) tropomyosin and can induce basophil histamine release.^6^ Some *Schistosoma mansoni* (*Sm*) venom allergen-like (SmVAL) proteins (with orthologues in wasps) and tegumental allergen-like (SmTAL) proteins have allergenic properties.^7,8^ Recognition of such phenomena has provided insights regarding epidemiological trends of allergy in the tropics.^9^

The role of carbohydrate cross-reactivity in epidemiology of allergic sensitization and disease is less understood. Among the commonest sources of cross-reactivity between natural allergens and other environmental exposures are specific asparagine (N) linked glycan modifications found on some helminth, insect and plant proteins (termed cross-reactive carbohydrate determinants, CCDs). The N-glycan trimannosyl-chitobiose core (Man_3_GlcNAc_2_) is conserved in eukaryotes. “Classical” CCD N-glycans expressed by invertebrate and plant proteins,^10,11^ and on antigenic surfaces and in secretomes of schistosome eggs^12^ and some nematodes,^13–16^ carry additional, nonmammalian, IgE-binding motifs: 1) β-(1,2) xylose linked to the first mannose of the trimannosyl component and/or 2) α-(1,3) fucose linked to the asparagine-linked N-acetylglucosamine (GlcNAc) of the glycan core.^17,18^ Glycoproteins carrying such N-glycans are potent immune determinants, inducing strong Th2-type responses,^23^ and comprising epitopes for antibodies, including IgE.^28–30^ However, the relevance of these glycan motifs in allergy-related disease is unclear. Barring a few exceptions,^31–33^ anti-CCD IgE seems to have poor biological function: individuals with specific IgE to CCDs lack skin and oral reactivity to the same molecules.^10,29,34^

In the tropics, CCDs have been reported to dominate specific IgE responses.^4^ Among Ghanaian schoolchildren, peanut-IgE-sensitization assessed by ImmunoCAP^®^ was prevalent and strongly associated with anti-CCD IgE and *Schistosoma haematobium* infection, but not clinical peanut allergy.^35^ The separation of anti-CCD IgE responses from clinical allergy symptoms, coupled with abundance of immunogenic CCDs on glycoproteins from environmental antigens prevalent in tropical settings (such as some schistosome antigens), led us to hypothesize an inverse association between anti-CCD IgE reactivity and clinical allergy, possibly indicative of CCD-mediated inhibition of allergic effector responses.

We conducted three studies in varied Ugandan settings to obtain a comparative assessment of allergy-related disease prevalence and risk factors in rural^36^ and urban^37^ settings, and among asthmatic children and controls.^38^ These studies provide an unprecedented opportunity to assess anti-CCD IgE profiles, their relevance to epidemiological trends of allergic sensitization and asthma, and their association with the rural-urban environment and *Sm* exposure in tropical LICs.

## 2 Methods

### 2.1 Studydesignandpopulation

The three studies comprised rural and urban cross-sectional surveys on allergy outcomes and a case-control study on asthma risk factors among schoolchildren in Uganda.

The rural survey (September 2015-August 2016) was conducted in *Sm*-endemic fishing villages of Koome islands, Lake Victoria, Uganda (Figure S1). It was the three-year outcome survey of the Lake Victoria Island Intervention Study on Worms and Allergy-related diseases [LaVIISWA; ISRCTN47196031^39^], a cluster-randomized trial of community-wide standard versus intensive anthelminthic treatment.^39,40^ The urban survey (September 2016-September 2017) was conducted in Entebbe municipality, a lower helminth exposure, urban setting^37^ situated on northern shores of Lake Victoria (Figure S1).

The asthma study (May 2015-July 2017)^38^ enrolled children with doctor-diagnosed asthma and controls from schools in Entebbe and surrounding areas. All schoolchildren were screened using the International Study on Allergy and Asthma in Children (ISAAC) questionnaire.^41^ Children with a history of wheeze in the last year underwent a detailed clinical assessment by study clinicians, including a medical and treatment history, lung function tests, and were assessed for asthma control using the Asthma Control Test.^38,42^ Among cases, 55.5% had well-controlled asthma, 29.5% partly controlled and 15% poorly controlled asthma. Only three cases had abnormal lung function tests. All cases were seen at one time point, started on the recommended treatment and were referred for additional management.^42^ Children in the same class as cases, with no history of wheezing, were eligible as controls. A Stata program (College Station, Texas, USA) was used to randomly select participants from each class register such that the number of controls was twice the number of cases.

Recent wheeze was also assessed in the rural and urban surveys using ISAAC questionnaires. Other procedures were identical in all three studies, including assessment of skin prick test (SPT) reactivity to allergens common in our setting^43^: *Dermatophagoides* mix (*Dpteronyssinus and D farinae*), *Blomia tropicalis* and *Blattella germanica* (ALK-Abelló; supplied by Laboratory Specialities [Pty] Ltd., South Africa). Visible flexural dermatitis [evaluated following Williams’ online manual^44^], and questionnaire-determined recent rhinitis and urticarial rash were also assessed.

### 2.2 Parasitological examinations

Infection with *Sm* and intestinal helminths was investigated using the Kato-Katz technique^45^ conducted on one stool sample per participant (two slides, read by different technicians). In the urban and rural surveys, stool was further examined for *Sm, Necator americanus* and *Strongyloides stercoralis* infections using PCR,^46,47^ and urine for *Sm* circulating cathodic antigen (CCA, Rapid Medical Diagnostics, South Africa).

### 2.3 Measurement of allergen-and glycan-specific IgE

The ImmunoCAP^®^ specific IgE test [Thermo Fisher Scientific, Uppsala, Sweden] (hereinafter “ImmunoCAP^®^) was used to measure plasma IgE levels to whole allergen extracts of house dust mite (*D pteronyssinus*, HDM), peanut *(Arachis hypogaea)* and German cockroach (*B germanica)*, with ≥ 0.35 kU/L defining allergic sensitization.^48^ The ImmunoCAP^®^ method is described in this article’s Supplementary information.

To assess IgE reactivity to established major allergenic components of these and other extracts, the ISAC (Immuno Solid-phase Allergen Chip) microarray [Thermo Fisher Scientific]^49–51^ was used. The ISAC microarray comprised112 allergen components from 51 sources. The binding assay is described in this article’s Supplementary information. The array comprises 67 recombinant components [produced in *E coli*, hence nonglycosylated^52^]; and 45 components purified from natural extracts (denoted by prefix “n”, eg nMUXF3 for the glycan epitope from bromelain). Previous work has shown that nMUXF3 and glycoproteins from pollen (nPhl p 4, nCyn d 1, nPla a 2, nCry j 1, nCup a 1) and food (nJug r 2) are recognized by anti-CCD IgE, while other natural allergens on the ISAC are not.^53^ Herein, “CCD-bearing components” denotes ISAC components confirmed to carry the classical core β-1,2-xylose and/or α-1,3-fucose substituted N-glycans. We also use the term “established major allergenic components” to refer to specific proteins that bind IgE to their protein backbone and are well-recognized as the molecular drivers of allergenic potency, in specific allergen sources.^54,55^ Participants with an ISAC standardized unit (ISU) measurement of ≥ 0.3 were considered sensitized to that allergen component.^56,57^ Measurements were also reported as detectable versus undetectable (lower detection limit: 0.06 ISU).

A noncommercial microarray of 135 chemo-enzymatically synthesized glycans with and without β-1,2-xylosylation and/or α-1,3-fucosylation (Figure S2) was employed to measure plasma anti-glycan IgE. Microarray construction procedures have been published.^58,59^ The IgE-binding assay and the microarray image processing procedures were adapted from previous studies^27,60–62^ and are detailed in the Supplementary information. Median fluorescence intensities (MFIs) reported herein represent anti-glycan IgE concentrations, because fluorescence-labelled anti-human IgE was used to detect plasma IgE bound to individual glycans on the array.

Samples were also assessed for *Schistosoma* egg [SEA]- and adult worm [SWA] antigen-specific IgE, IgG and IgG4, by ELISA [described elsewhere^63^].

### 2.4 Statistical analysis

Statistical analyses were done in Stata 13.1, GraphPad Prism 7.0a (Fay Avenue, CA, USA) and R via the RStudio interface (version 1.1.383; Boston, USA). Initial analyses in the rural survey investigated the impact of trial intervention on IgE profiles using a cluster-level approach, as previously described.^36^ Differences in characteristics between rural and urban survey participants and between asthmatic and nonasthmatic children were assessed using logistic or linear regression, allowing for the survey designs (clustering and weighting). Unadjusted analysis of differences in individual N-glycan-/allergenspecific IgE levels between rural and urban participants, and between asthmatics and controls was done using the Mann-Whitney U test, correcting for multiple testing using a Monte Carlo simulation approach^64^ with 1000 permutations, to generate empirical p values. For analyses comparing prevalences of ISAC-determined IgE sensitization between these groups, chi-squared tests (or Fisher’s exact test, for expected cell counts < 5) were used.

Since anti-glycan IgE responses were strongly correlated, they were further analysed using principal component analysis (PCA). Data for participants from all studies were pooled and principal component (PC) scores generated. Unadjusted and age- and sex-adjusted associations between PC scores and atopic sensitization, asthma status, *Sm* infection and survey setting were assessed using linear regression.

## 3 Results

### 3.1 Participants’ characteristics

**Figure 1:**
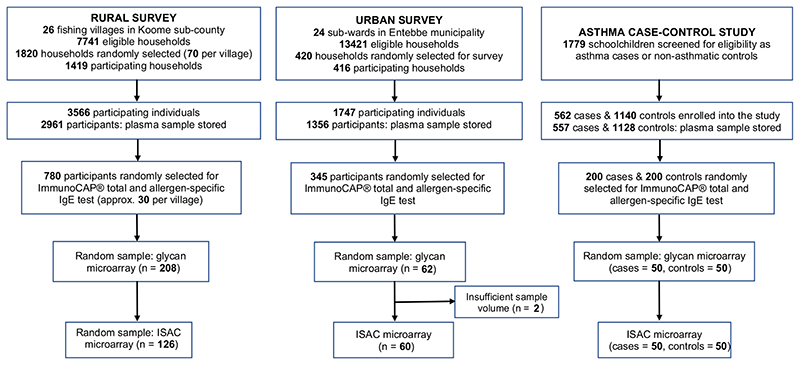
Selection of samples for the ImmunoCAP^®^ test and the ISAC and glycan microarray assays

Allergen- and glycan-specific IgE measurements were conducted in a subset of randomly selected samples per study (Figure 1). Data on ImmunoCAP^®^-determined IgE sensitization were available for 780, 345 and 400 participants of the rural survey, urban survey and asthma study, respectively. For the rural survey, we present data combined from both anthelminthic treatment arms, because there was no effect of trial arm on ImmunoCAP^®^- or ISAC-determined IgE sensitization or on anti-glycan IgE reactivity (Table S1).^36^

Table 1 shows participants’ characteristics. Rural participants were on average older than urban participants (*P* = .001) and more likely to be male. SPT reactivity to *Dermatophagoides* mix (*P* = .003) and *B tropicalis* (*P* = .025) was higher among urban participants, while total IgE (*P* < .001) and allergen extract-specific IgE sensitization (ImmunoCAP^®^ concentration ≥ 0.35 kU/L), particularly to cockroach (*P* < .001), was higher among rural participants. Urticarial rash was more common in rural participants (*P* < .001), while wheeze, rhinitis and dermatitis were rare in both settings. Rural, compared to urban participants, was more frequently infected with *Sm* (*P* < .001), *T trichiura* (*P* = .002), hookworm (*P* = .016) and *S stercoralis* (*P* = .001) and had higher median levels of SEA- and SWA-specific IgE, IgG and IgG_4_ (*P* < .001). Adjusting for age and sex did not impact these differences.

Asthmatics, compared to nonasthmatic controls, had higher prevalence of SPT reactivity, ImmunoCAP^®^ IgE sensitization, dermatitis and rhinitis, and higher total IgE levels (Table 1). ImmunoCAP^®^ IgE sensitization was linked to exposure-related asthma symptoms. For example, of the children who reported dust as a trigger for their asthma symptoms, 65.6% were sensitized (IgE ≥ 0.35 kU/L) to *D pteronyssinus*, compared to 46.7% who did not report dust as a trigger (*P* = .009). This difference was not statistically significant for *B germanica* and *A hypogaea-specific* IgE sensitization.

Prevalence of helminth infections in the asthma study was low, but concentrations of SEA- and SWA-specific antibodies were moderate, and similar between asthmatics and nonasthmatics (Table 1).

### 3.2 Overall responses to structures on the ISAC and N-glycan microarray

Figure S3 shows prevalence of sensitization (IgE ≥ 0.3 ISU) to allergen components on the ISAC microarray. Among rural participants, sensitization to components on the array was dominated by reactivity to natural components bearing classical CCDs and to insect venom proteins (rPol d 5, rVes v 5, rApi m 1). Among urban participants and nonasthmatics, sensitization patterns were more varied: natural and/or recombinant HDM, venom, food and CCD-bearing components contributed most to sensitization. However, sensitization among asthmatics was dominated by reactivity to major recombinant and natural HDM allergens.

Figure S4 shows average MFIs for structures on the glycan microarray. Although the highest responses were raised predominantly against core β-1,2-xylose and/or α-1,3-fucose substituted N-glycans in all three studies, responses to other carbohydrate structural elements represented in the array, such as tri-mannose (G99), fucosylated GlcNAc (G111) and galactose-alpha-1,3-galactose (α-1,3-gal) [G112, G113], were also as high.

### 3.3 Sensitization to allergen extracts does not reflect sensitization to their established major allergenic components, except among asthmatics

In all study settings, sensitization to HDM, German cockroach and peanut extracts (ImmunoCAP^®^ IgE ≥ 0.35 kU/L) was high (Table 1). Fifty five per cent and 43% of rural and urban participants and 73% and 54% of asthmatic schoolchildren and their controls, respectively, were sensitized to at least one of the three extracts. Prevalence of SPT reactivity to each extract was lower than prevalence of IgE sensitization to the extracts of the same allergens (Table 1). The majority of SPT reactive individuals (88% rural, 90% urban, 95% asthmatic and 93% nonasthmatic controls) were also IgE-sensitized to at least one of the three extracts on ImmunoCAP (data not shown).

**Figure 2:**
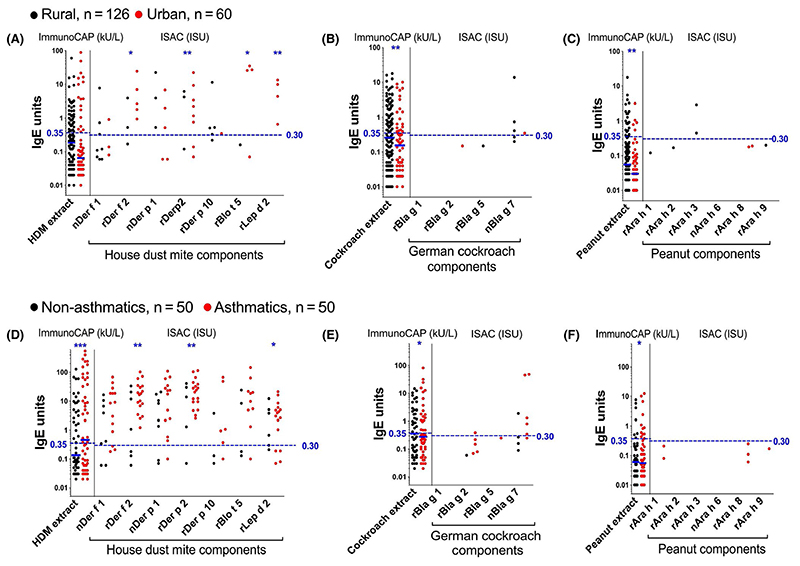
IgE reactivity to allergen extracts and their major allergenic components. ImmunoCAP^®^-determined IgE sensitization to HDM, German cockroach and peanut extracts and ISAC-determined IgE sensitization to the major allergenic components in these extracts. Natural and recombinant allergens on the ISAC array are denoted by the prefixes (n) and (r), respectively, on the allergen name. Figure shows data points from individuals with detectable IgE levels. Cut-offs of 0.35 kU/L and 0.30 ISU are shown to define the levels of ImmunoCAP^®^ and ISAC responses, respectively, that are important for clinical diagnosis of allergic sensitization. The Mann-Whitney *U* test was conducted within the framework of a Monte Carlo simulation algorithm based on 1000 permutations (in order to adjust for multiple testing), to assess differences between rural and urban individuals (panels A, B and C), and between asthmatics and their controls (panels D, E and F): *P < .05; **P < .01; ***P < .001. HDM, house dust mite; ISAC, Immuno Solid-phase Allergen Chip; ISU, ISAC standardized units

However, ISAC microarray showed that sensitization to both natural (non-CCD-bearing) and recombinant forms of established major allergenic components of these extracts was very low among rural and urban survey participants, and among nonasthmatic schoolchildren (Figure 2): 0%-3% (rural), 0%-10% (urban) and 0%-12% (nonasthmatics) of tested participants were sensitized (IgE ≥ 0.30 ISU) to HDM, cockroach and peanut components on ISAC. The picture was strikingly different among asthmatics: 18%-36% were sensitized to HDM allergens (42% to at least one) and 0%-10% to cockroach, but none to peanut components.

### 3.4 Reactivity to CCDs dominates ISAC-determined IgE profiles

**Figure 3:**
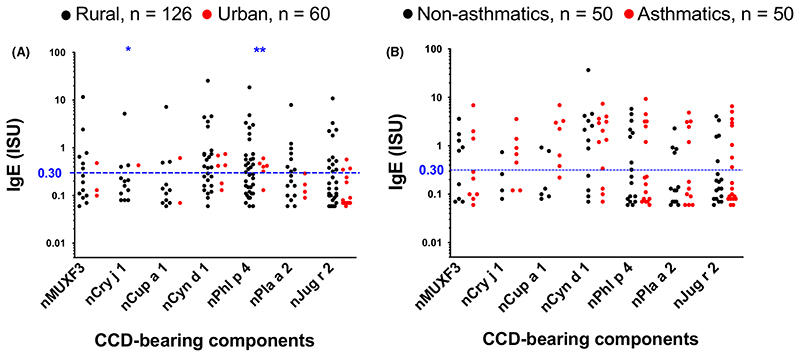
ISAC microarray-determined IgE reactivity to components carrying classical CCDs. Natural and recombinant allergens are denoted by the prefixes (n) and (r), respectively, on the allergen name. Figure shows data points from individuals with detectable IgE levels. Cut-offs of 0.30 ISU are shown to define the level of ISAC-determined IgE responses that is important for clinical diagnosis of allergic sensitization. The Mann-Whitney *U* test was conducted within the framework of a Monte Carlo simulation algorithm based on 1000 permutations (in order to adjust for multiple testing), to assess differences between rural and urban individuals, and between asthmatics and their controls: *P < .05; **P < .01; ***P < .001. ISU, ISAC standardized units; CCD, cross-reactive carbohydrate determinant

Despite the low prevalence of ISAC-determined IgE sensitization to major natural and recombinant components of common allergens in our study settings, many participants mounted responses to components bearing classical CCDs (Figure 3A and B). A higher proportion of rural, compared to urban participants, recognized CCDs (Table S2 and Figure 3A). However, this was statistically significant only for the CCD-bearing components nPhl p 4 and nCry j 1. Conversely, a higher proportion of urban, compared to rural participants, recognized recombinant established major allergens of HDM (Table S2 and Figure 2A), the food components rApi g 1 and rMal d 1 and the cat allergen rFel d 4 (Table S2).

Although reactivity to HDM components was higher among asthmatics compared to controls, reactivity to components bearing classical CCDs and most other allergen components on the array was similar between the two groups (Figure 2 and Table S2).

Many participants also mounted responses to venom proteins (Figure S5). This may represent insect venom sensitization; however, higher reactivity was observed among rural compared to urban participants (Table S2 and Figure S5) and among *Sm* infected compared to uninfected participants (Table S3), possibly reflecting sensitization to Venom-Allergen-Like (VAL) proteins expressed by schisto-somes^65^ and other helminths.^66,67^

### 3.5 Reactivity to core β-1,2-xylose/α-1,3-fucose is positively associated with *Sm* infection and sensitization to allergen extracts, but not sensitization to their established major allergenic components

Cross-reactive carbohydrate determinants expressed by several insect and plant glycoproteins, and by some nematodes and trematodes (such as schistosomes), are typified by presence of α-1,3-linked core fucose and β-1,2-linked core xylose motifs.^11,18^ We combined the three studies and further explored associations between CCD-specific IgE and *Sm* infection and atopic sensitization using microarray binding studies of core β-1,2-xylose and α-1,3-fucose substituted N-glycans.

**Figure 4:**
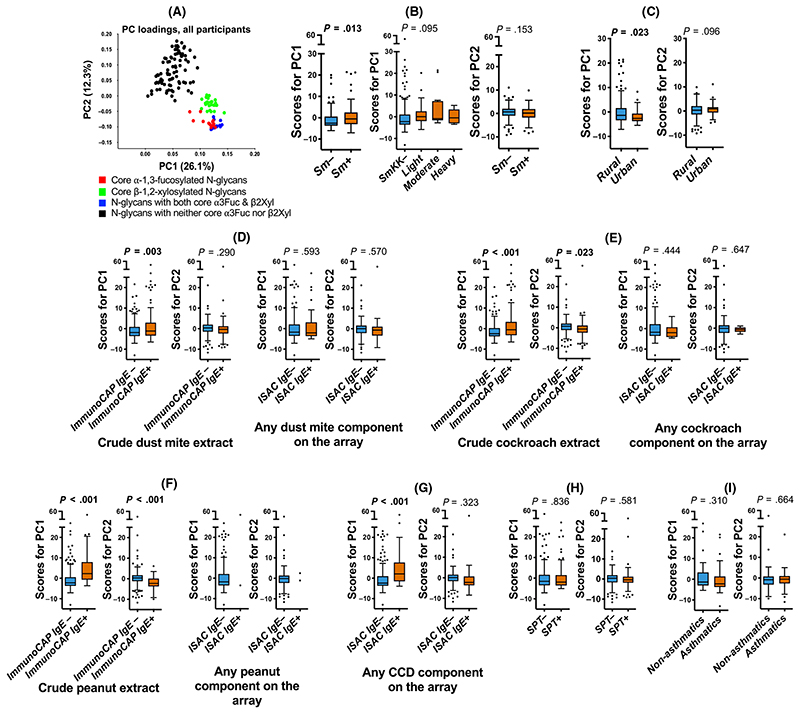
Associations between anti-glycan IgE responses and atopic sensitization and asthma. Figure shows data combined from all three studies. Panel (A) shows a scatterplot of first (PC1) and second factor (PC2) loadings derived from principal component analysis of IgE responses to 135 synthetic N-glycans. Box-and-whisker plots show associations between PC scores and (B) S. mansoni infection [Kato-Katz and/or PCR, infected n = 88; uninfected n = 154], (C) setting [rural n = 208; urban n = 62], (D) IgE sensitization (ImmunoCAP^®^ IgE ≥ 0.35 kU/L) to crude D. pteronyssinus extract [sensitized n = 145; nonsensitized n = 224] and sensitization (IgE ≥ 0.3 ISU) to any of the dust mite components on the ISAC array [sensitized n = 47; nonsensitized n = 239], (E) IgE sensitization to crude German cockroach extract [sensitized n = 167; nonsensitized n = 201] and to any of the German cockroach components on the ISAC array [sensitized n = 11; nonsensitized n = 275], (F) IgE sensitization to crude peanut (*A. hypogaea*) extract [sensitized n = 55; nonsensitized n = 314] and to any of the peanut components on the ISAC array [sensitized n = 2; nonsensitized n = 284], (G) IgE sensitization (IgE ≥ 0.30 ISU) to any of the components bearing classical CCDs [sensitized n = 47; nonsensitized n = 239], on the ISAC array, (H) SPT reactivity to any of Dermatophagoides mix, German cockroach or Blomia tropicalis [SPT + n=125; SPT- n = 241] and (I) asthma status (asthmatic n = 50; nonasthmatic n = 50). Horizontal lines in the plots represent medians and boxes denote interquartile ranges (IQR). Whiskers were drawn using the Tukey method (1.5 times IQR). Individual points represent outliers (>1.5 times IQR away from median). Associations between PC scores and the various comparison groups were assessed using linear regression analysis in Stata 13.1. Age- and sex-adjusted *P* values are shown. PC1, principal component 1; PC2, principal component 2; Sm, *S. mansoni* infection determined by Kato-Katz and/or PCR; ISAC, Immuno Solid-phase Allergen Chip; CCD, cross-reactive carbohydrate determinant; SPT, skin prick test

Responses to individual core substituted N-glycans were strongly correlated and were combined using PCA. Figure 4A shows scatterplots of PC1 and PC2 loadings (all participants). PC1 was characterized by responses to core β-1,2-xylose and/ or α-1,3-fucose substituted N-glycans while PC2 was characterized by responses to nonxylosylated and nonfucosylated glycans. There were positive associations between PC1 scores and (a) *Sm* infection (Figure 4B) [but not other helminths, data not shown], (b) the rural environment (Figure 4C), (c) HDM, cockroach and peanut extract-sensitization (on ImmunoCAP^®^) and (d) CCD sensitization [on ISAC] (Figure 4D–G). However, no associations were observed between PC1 scores and sensitization to any of the major natural and recombinant HDM, cockroach and peanut components on ISAC (Figure 4D–F).

### 3.6 Inverse associations between reactivity to a subset of core α-1,3-fucosylated N-glycans and asthma, but not for CCD-specific IgE in general

**Figure 5:**
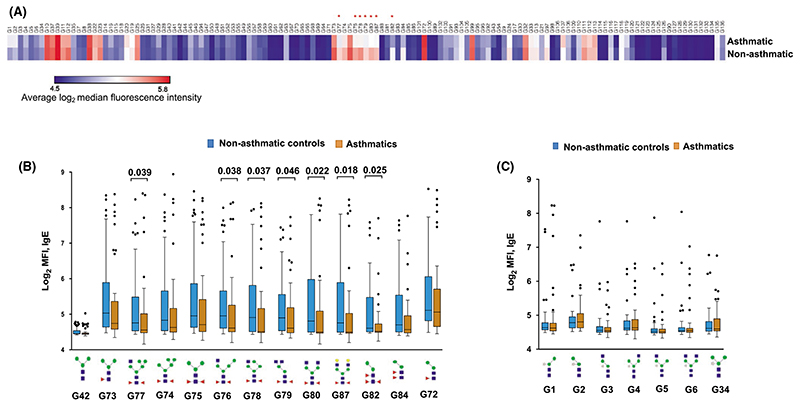
Associations between asthma and IgE reactivity to glycans. (A) Heatmap of average log_2_-transformed MFIs representing IgE reactivity to each individual glycan structure on the array, in asthmatic (n = 50) versus nonasthmatic schoolchildren (n = 50). *P < .05. (B) Box-and-whisker plots showing MFIs representing IgE reactivity to core α-1,3-fucosylated N-glycan structures. (C) Box-and-whisker plots showing MFIs representing IgE reactivity to core β-1,*2-xylosylated* N-glycan structures. Horizontal lines in the plots represent medians, and boxes denote interquartile ranges (IQR). Whiskers were drawn using the Tukey method (1.5 times IQR). Individual points represent outliers (> 1.5 times IQR away from median). The Mann-Whitney U test was conducted within the framework of a Monte Carlo simulation algorithm based on 1000 permutations (in order to adjust for multiple testing), to assess differences between asthmatic and nonasthmatic schoolchildren

No associations were observed between glycan microarray-assessed IgE response PC scores and asthma or SPT reactivity (Figure 4H–I). Assessment of associations between asthma and responses to individual glycans on the array showed that asthmatics, compared to controls, mounted significantly lower responses to N-glycans carrying core α-1,3-fucose only or in combination with α-1,6-fucose, but not to any other structures on the array (Figure 5).

We did not group asthma cases into “allergic” and “nonallergic” based on ImmunoCAP^®^ IgE sensitization, as our data showed that IgE to allergen extracts reflected sensitization to environmental exposures such as CCDs, and could not accurately define allergic asthma. However, we grouped asthma cases based on SPT reactivity to at least one allergen extract, to assess associations with responses to N-glycans carrying core α-1,3-fucose (Figure S6A). Compared to nonasthmatic controls, both “allergic” (SPT+) and “nonallergic” (SPT-) asthmatics mounted lower responses to core α-1,3-fucose substituted N-glycans. There were no differences in responses between SPT- and SPT + asthma cases. Similar results were observed when we based on sensitization to at least one recombinant allergen component on the ISAC microarray to group asthma cases into “allergic” (IgE ≥ 0.3 ISU) and “nonallergic” (IgE < 0.3 ISU) [Figure S6B].

## 4 Discussion

We conducted an assessment of associations between IgE responses at (sub-)molecular level and allergy outcomes among Ugandan rural and urban individuals, and asthmatic schoolchildren and their controls. We found that among rural, urban and nonasthmatic participants, sensitization to extracts from common environmental allergens did not reflect sensitization to their established major allergenic components; rather, many participants recognized CCDs. By contrast, a high proportion of asthmatics were sensitized to both HDM extract and its major components, lending substantial support to increasing evidence that much asthma in tropical LICs is atopic, contrary to earlier perception.^68,69^ Detailed assessment of IgE reactivity to core β-1,2-xylose and/or α-1,3-fucose (well-known, nonmammalian, “classical” CCDs) using a synthetic glycan microarray revealed strong positive associations with the rural environment and *Sm* infection and for the first time showed reactivity to a subset of core α-1,3-fucose substituted N-glycans to be negatively associated with asthma.

Positive associations between antibody reactivity to classical CCD epitopes and the rural environment and *Sm* infection among our urban and rural survey participants were initially reported in our recent publication.^70^ Here we present data from an allergen extract-based test (ImmunoCAP^®^) and an allergen (glyco)protein microarray (ISAC) to confirm these associations and to explore associations with allergic sensitization and asthma.

Allergen extracts contain a mixture of allergenic and nonaller-genic components,^71^ as well as cross-reactive protein and carbohydrate components that are conserved in other environmental antigens such as those from schistosomes.^72^ This obscures the identity of the molecular drivers, and hence interpretation, of a positive response in vitro to extract-specific IgE blood immunoassays (or in vivo to allergen extract-based SPTs). Our results show that this has important implications for diagnosis of atopy, and phenotyping of asthma (and other allergy-related conditions) as “atopic”, in tropical settings and for the use of population attributable fractions to assess the contribution of atopy to allergy-related disease in tropical LICs.

The high prevalence of sensitization to CCDs and to allergen extracts but low sensitization to established major allergenic components in our high-*Sm*-transmission rural setting is reminiscent of previous findings by Hamid *et al* among Indonesian schoolchildren from areas endemic for soil-transmitted helminths (STH) but not schistosomes.^4^ These results imply that cross-reactive protein motifs and/or CCDs from environmental antigens other than schistosomes play an important role in allergy epidemiology. Exposure to STH might theoretically contribute; however, as discussed in our previous article on associations between helminth infections and N-glycan-reactivity,^70^ expression of “classical” CCD epitopes in most STH is not yet well mapped, with STH glycomes understudied compared to the *Schistosoma* glycome. However, absence of significant associations between anti-CCD IgE and infection with any STH (reported here and in our previous study^70^) opposes the notion that STH express “classical” CCDs.

There is evidence showing that cross-sensitization between *Schistosoma* antigens and peanut, pollen or insect venom allergens is predominantly caused by CCDs.^35,73,74^ It is likely that previously observed positive associations between *Sm* and HDM extract-specific IgE in our rural setting^40^ are partially attributable to CCD sensitization. In the same population, prevalence of clinical allergies is low, supporting the hypothesis that helminths may protect against allergic effector responses. The role of CCDs in protection against allergy, if any, is unclear. Alpha-1,3-gal has been implicated in meat allergy^33^; however, our studies did not find any evidence of this, despite elevated α-1,3-gal-specific IgE concentrations. Other than α-1,3-gal-specific IgE, carbohydrate-specific IgE rarely, if ever, translates into clinical allergy.^10,29,34,35^ We postulated that elevated IgE responses to specific immunogenic glycans during chronic *Sm* infection might result in reduced allergic effector responses. Many native allergens occur as glycoproteins; therefore, prior high exposure to specific CCDs (perhaps resulting from *Sm* infection or other exposures) may prime initial and recall IgE responses to preferentially target-specific CCD rather than the protein epitopes of allergens. Indeed, rural-urban comparisons of responses to components on the ISAC array imply that in our *Sm*-endemic rural setting, IgE may be less effectively induced against common protein allergens than against CCDs.

In our setting, ISAC-determined anti-CCD IgE was not inversely associated with SPT reactivity or asthma, and PCA of microarray-determined anti-glycan IgE responses suggested no overall association (inverse or otherwise) between asthma and anti-glycan IgE. However, analysis of levels of IgE to individual glycans revealed that reactivity to a subset of core α-1,3-fucose substituted N-glycans was lower among asthmatic schoolchildren compared to controls, and this was consistent for all N-glycan structural variants on the array bearing core α-1,3-fucose only or in combination with α-1,6-fucose. It is plausible that responses to specific glycans are not merely a smokescreen that obscures readouts for assessment of atopy. Mechanistic investigations in animal and human studies are required to investigate whether specific glycans might be involved in protection against asthma, or whether they merely represent surrogate markers of protective environmental exposures. Current efforts to reconstruct specific glycoproteins (including those carrying N-glycan core substitutions) in plant systems^75^ offer great promise for availability of tailored glycan epitopes for initial testing in animal models. The observed inverse associations between asthma and core α-1,3-fucosylated N-glycans are unlikely to represent an “epiphenomenon” (merely reflective of inverse *Sm*-asthma associations) since, from our data, *Sm* prevalence and concentrations of SEA- and SWA-specific antibodies were similar between asthma cases and their controls.

Our study had some limitations. First, it would have been valuable to have a comparison group of asthmatics from the rural setting, to ascertain the generalizability of our findings and their interpretation. However, as shown in Table 1 and in our previous assessments,^37^ the prevalence of wheeze (considered a good proxy for asthma in epidemiological studies^76^) in our rural setting was very low (and some of it probably nonasthma related). Second, the urban community had considerable exposure to light helminth infections, while rural participants had received three years of mass anthelminthic treatment at the time of the survey. Nonetheless, the two settings were still quite distinct with regard to helminth prevalence, providing an interesting sample for assessing urban-rural differences in fine specificities of IgE responses to allergen extracts, their major allergenic components and “classical” CCD motifs.

Overall, our data indicate that, in tropical LICs, IgE to allergen extracts (detected in standard ImmunoCAP^®^ assays) reflects sensitization to a myriad of environmental exposures (absent in high-income countries), such as CCDs, and does not accurately define allergy-related disease phenotypes. In fact, our findings call for studies to investigate whether specific CCD epitopes have a mechanistic role in the protective effect (against asthma) of certain environmental exposures.

## Supplementary Material

Additional supporting information may be found online in the Supporting Information section.

Supplementary information

## Figures and Tables

**Table 1:** Characteristics of study participants

Characteristics	Rural survey	Urban survey	*P^b^*	Case-control study on asthma in schoolchildren n/N (%)		*p*
	n/N (%)^a^	n/N (%)^a^		Nonasthmatics	Asthmatics	
Socio-demographic						
Age in years, median (IQR)	**28 (21, 36)**	22 (10, 32)	**.001**	10 (8, 13)	**11 (10, 14)**	**.017**
Male sex	**377/780 (46.4)**	97/345 (28.1)	**<.001**	94/200 (46.7)	92/200 (46.0)	.841
Allergy-related outcomes						
Skin prick test reactivity						
Any	164/780 (21.3)	72/336 (21.4)	.955	60/198 (30.3)	**105/198 (53.0)**	**<.001**
*Dermatophagoides* mix	87/780 (10.0)	**61/336 (18.2)**	**.003**	47/198 (23.7)	**91/198 (45.9)**	**<.001**
*Blomia tropicalis*	54/780 (6.8)	**41/336 (12.2)**	**.025**	45/198 (22.7)	**90/198 (45.4)**	**<.001**
*Blattella germanica*	98/780 (13.9)	45/337 (13.4)	.811	32/198 (16.2)	**48/198 (24.2)**	**.046**
asIgE sensitization (≥0.35 kU/L, ImmunoCAP)						
Any	**437/780 (55.1)**	148/345 (42.9)	**.007**	108/200 (54.0)	**145/199 (72.9)**	**<.001**
*D pteronyssinus*	264/780 (33.2)	104/345 (30.1)	.421	72/200 (36.0)	**117/200 (58.5)**	**<.001**
*Blattella germanica*	**393/780 (49.8)**	118/345 (34.2)	**<.001**	90/200 (45.0)	**112/199 (56.3)**	**.025**
*Arachis hypogaea*	114/780 (14.9)	41/345 (11.9)	.266	24/200 (12.0)	**39/200 (19.5)**	**.041**
asIgE concentration (kU/L, ImmunoCAP), median (IQR)						
*D pteronyssinus*	0.2 (0.0, 0.6)	0.1 (0.0, 0.7)	.229	(0.0, 1.9)	**1.0 (0.1, 37.5)**	**<.001**
*Blattella germanica*	0.4 (0.1, 1.5)	0.1 (0.0, 0.6)	.949	0.3 (0.1, 1.1)	**0.6 (0.1, 2.9)**	**<.001**
*Arachis hypogaea*	0.1 (0.0, 0.2)	0.0 (0.0, 0.1)	.877	0.1 (0.0, 0.2)	0.1 (0.0, 0.2)	.101
Total IgE (kU/L, ImmunoCAP), median (IQR)	**672 (250, 1942)**	159 (57, 523)	**<.001**	279 (98, 648)	**487 (115, 1248)**	**.018**
Wheeze in last 12 mo, age < 5 y	1/58 (0.9)	2/37 (5.4)	.188			
Wheeze in last 12 mo, age ≥ 5 y	24/716 (2.9)	5/272 (1.8)	.308	0/200 (0.0)	200/200 (100.0)	
Visible flexural dermatitis	4/780 (0.5)	3/345 (0.9)	.462	3/199 (1.5)	**13/198 (6.6)**	**.019**
Rhinitis in last 12 mo	34/774 (4.2)	11/309 (3.6)	.700	11/199 (5.5)	**43/198 (21.7)**	**<.001**
Urticaria in last 12 mo	**98/773 (12.4)**	11/309 (3.6)	**<.001**	5/199 (2.5)	6/198 (3.0)	.754
Helminth infections						
*S mansoni* (KK)	**187/679 (29.5)**	14/284 (4.9)	**<.001**	8/194 (4.1)	12/184 (6.5)	.302
*S mansoni* intensity (KK)						
Uninfected	492/679 (70.5)	**270/284 (95.1)**		186/194 (95.9)	172/184 (93.5)	
Low	**94/679 (14.7)**	7/284 (2.5)		4/194 (2.1)	7/184 (3.8)	
Moderate	**53/679 (8.9)**	4/284 (1.4)		3/194 (1.5)	3/184 (1.6)	
Heavy	**40/679 (5.8)**	3/284 (1.1)	**<.001**	1/194 (0.5)	2/184 (1.1)	.328
***S mansoni* (urine CCA)** ^c^	**590/724 (82.4)**	108/309 (34.9)	**<.001**			
***S mansoni* (PCR)** ^c^	**310/679 (47.5)**	43/282 (15.3)	**<.001**			
***A lumbricoides* (KK)**	2/679 (0.2)	0/284 (0)		0/194 (0)	1/184 (0.5)	
***T trichiura* (KK)**	**44/679 (6.2)**	4/284 (1.4)	**.002**	4/194 (2.1)	2/184 (1.1)	.456
***N americanus* (PCR)**	**72/679 (9.9)**	12/282 (4.3)	**.016**	3/194 (1.6)^d^	2/184 (1.1)^d^	.697
***S stercoralis* (PCR)** ^c^	**58/679 (7.5)**	4/282 (1.4)	**.001**			
*Schistosoma-*specific antibody levels (μg/mL), median (IQR)						
SEA-specific IgE	**4.6 (3.1, 6.6)**	2.6 (1.7, 4.4)	**<.001**	2.4 (1.5, 4.0)	2.4 (1.7, 3.9)	.566
SWA-specific IgE	**4.9 (3.0, 6.7)**	2.3 (1.5, 3.4)	**<.001**	1.9 (1.3, 3.4)	2.0 (1.5, 3.3)	.914
SEA-specific IgG_4_	**282 (70, 839)**	27 (0, 90)	**<.001**	27 (0, 77)	26 (0, 68)	.255
SWA-specific IgG_4_	**109 (52, 275)**	39 (18, 65)	**<.001**	38 (22, 64)	39 (22, 60)	.871
SEA-specific IgG	**1975 (1061, 3098)**	739 (599, 1476)	**<.001**	736 (602, 1318)	693 (593, 1117)	.686
SWA-specific IgG	**1499 (999, 2140)**	791 (612, 1200)	**<.001**	825 (644, 1130)	737 (615, 1061)	.495

*Note:* Table shows characteristics for individuals with data on ImmunoCAP^®^-determined IgE sensitization. Probability values are shown for differences in characteristics between rural and urban survey participants and between asthmatic schoolchildren and nonasthmatic controls.

Abbreviations: asIgE, allergen-specific IgE; CCA, circulating cathodic antigen; IQR, interquartile range; KK, Kato-Katz; PCR, polymerase chain reaction; SEA, Schistosoma egg antigen; SWA, Schistosoma adult worm antigen.

a Percentages were adjusted for survey design. Percentages/medians that were significantly higher in one group compared to the other (*P* ≤ .05) are highlighted in bold. Adjusting for age and sex had little impact on these differences.

b P values obtained from survey design-based logistic or linear regression.

c Information not collected in the asthma case-control study.

d Necator americanus infection detected by Kato-Katz in the asthma case-control study.

## Abbreviation

CCD: cross-reactive carbohydrate determinant
