# Supplementary material for: Cross-reactive carbohydrate determinant-specific IgE obscures true atopy and exhibits α-1,3-fucose epitope-specific inverse associations with asthma
Source: Allergy. Author manuscript; Available in PMC 2021 Jun 8. (PMC7610925; doi:10.1111/all.14469)
Supplement: Supplementary information [file EMS126390-supplement-Supplementary_information.docx]

SUPPLEMENTARY INFORMATION FOR

**Cross-reactive carbohydrate determinant-specific IgE obscures true atopy and exhibits ⍺-1,3-fucose epitope-specific inverse associations with asthma**

Gyaviira Nkurunungi^1,2,^*, Harriet Mpairwe^1^, Serge A Versteeg^3^, Angela van Diepen^4^, Jacent Nassuuna^1^, Joyce Kabagenyi^1^, Irene Nambuya^1^, Richard E Sanya^1,5^, Margaret Nampijja^1^, Sonia Serna^6^, Niels-Christian Reichardt^6,7^, Cornelis H Hokke^4^, Emily L Webb^8^, Ronald van Ree^3,a^, Maria Yazdanbakhsh^4,a^, Alison M Elliott^1,2,a^

^1^Immunomodulation and Vaccines Programme, Medical Research Council / Uganda Virus Research Institute and London School of Hygiene and Tropical Medicine (MRC/UVRI and LSHTM) Uganda Research Unit, P.O. Box 49, Entebbe, Uganda

^2^Department of Clinical Research, London School of Hygiene and Tropical Medicine, WC1E 7HT London, United Kingdom

^3^Departments of Experimental Immunology and of Otorhinolaryngology, Amsterdam University Medical Centers (AMC), 1105 AZ Amsterdam, The Netherlands

^4^Department of Parasitology, Leiden University Medical Center, 2333 ZA Leiden, The Netherlands

^5^College of Health Sciences, Makerere University, P.O. Box 7072, Kampala, Uganda

^6^Glycotechnology Laboratory, Centro de Investigación Cooperativa en Biomateriales (CIC biomaGUNE), Paseo Miramón 182, 20014 San Sebastián, Spain

^7^Centro de Investigación Biomédica en Red en Bioingeniería, Biomateriales y Nanomedicina (CIBER-BBN), Paseo Miramón 182, 20014 San Sebastián, Spain

^8^MRC Tropical Epidemiology Group, Department of Infectious Disease Epidemiology, London School of Hygiene and Tropical Medicine, WC1E 7HT London, United Kingdom

***Correspondence**: Gyaviira Nkurunungi, MRC/UVRI and LSHTM Uganda Research Unit, P.O. Box 49, Plot 51-59 Nakiwogo Road, Entebbe, Uganda & London School of Hygiene and Tropical Medicine, Keppel Street, London WC1E 7HT, UK. Telephone: +256752279917. Email: [gyaviira.nkurunungi@mrcuganda.org](mailto:gyaviira.nkurunungi@mrcuganda.org)

**^a^**Authors contributed equally to this work

SUPPLEMENTARY INFORMATION TEXT

# Supplementary methods

## Sample size considerations

In the rural survey, 2961 participants from the 26 study villages had a plasma sample stored and hence provided the sampling frame for selection for the ImmunoCAP IgE test. 780 participants (approximately 30 per village) were randomly selected using Stata 13.1 software (StataCorp, College Station, Texas, U.S.A). The rural survey was the three-year outcome survey of the Lake Victoria Island Intervention Study on Worms and Allergy-related diseases (LaVIISWA; ISRCTN47196031 1). The main LaVIISWA trial analysis aimed to compare outcomes between the two trial arms,^1,2^ hence a sample size of 780 was expected to give 80% power to detect a 35% relative difference in the prevalence of ImmunoCAP allergen-specific IgE sensitisation between the two trial arms, based on an assumed overall allergen-specific IgE sensitisation prevalence of 50% (from results at baseline), and an estimated coefficient of variation (in IgE levels between clusters) of 0.2.

Samples for the glycan and ISAC microarray experiments were randomly selected from among those with ImmunoCAP data. We initially aimed for 50 samples per trial arm (100 in total), and managed to test a total of 209 (glycan array) and 126 samples (ISAC array experiments, respectively), owing to availability of further funding. Similar numbers have been shown to be useful in published studies that have assessed differences in microarray-assessed antibody levels between helminth infected and uninfected, and allergic and non-allergic individuals,^3,4^ and between children and adults.^5^

In the urban survey, 1356 participants had a plasma sample stored. ImmunoCAP data on cockroach- and dust mite-specific IgE were available for rural survey participants, hence calculations could be conducted to estimate how many urban survey plasma samples were required to attain significant differences in the prevalence of ImmunoCAP positivity between the urban and rural survey. Assuming a 35% prevalence of ImmunoCAP positivity to either cockroach or dust mite in the rural survey and a design effect of 1.3, 353 urban survey participants were required for 80% power to detect an absolute difference in proportion positive of 0.10 between the rural and urban survey. For the glycan and ISAC microarray experiments, a sample size of 50 was deemed sufficient to detect differences between the rural and urban setting, based on previous studies doing similar comparisons.^3^

In the asthma case-control study, 557 cases and 1128 controls had a plasma sample stored. Four hundred participants (200 asthmatics and 200 controls) were randomly selected for the ImmunoCAP test. This number was expected to yield 90% power to detect a significant positive association (at an odds ratio of 2) between asthma and ImmunoCAP positivity to any of dust mite, cockroach or peanut (IgE ≥0.35 kU/L). For glycan and ISAC microarray experiments, a sample size of 50 cases and 50 controls was deemed sufficient, based on numbers used in the rural and the urban survey.

### *Measurement of total IgE and allergen-specific IgE using the ImmunoCAP® test*

This test was conducted according to the manufacturer’s instructions. The ImmunoCAP® assay uses an automated system that loads serum / plasma on **1)** a hydrophilic polymer sponge (the ‘ImmunoCAP’) covalently coupled to an allergen (for specific IgE measurement) or **2)** an ImmunoCAP reaction vessel covalently coupled to an anti-IgE antibody (which reacts with total IgE in sample). Unbound IgE is washed off and a conjugate (anti-IgE antibody grafted with β-galactosidase) is added. The supernatant is then aspirated, and a substrate (4-methyl-umbelliferyl-β-D galactoside) deposited on the ImmunoCAP sponge. A stop solution is added and the sponge then compressed. The fluorescence generated by the resultant eluate, a product of enzyme degradation (4-methylumbelliferone, proportional to concentration of IgE in serum/plasma), is measured. Results are reported quantitatively using a kU/L scale. The calibrator is IgE bound to anti-IgE caps using a six-point quantitative curve. Calibration concentration ranges from 0 to 100 kU/L for specific IgE and 2 to 5000 kU/L for total IgE. A cut-off of 0.35 kU/L was used to define allergen-specific IgE sensitisation, as recommended by the test manufacturer.

## ImmunoCAP ISAC® sIgE 112 test

This test was conducted according to the manufacturer’s instructions. The ISAC (Immuno Solid-phase Allergen Chip) comprises four reaction sites, each printed with 112 allergen components in a microarray format. Before the immunoassay, each microarray chip was washed to remove any allergens that were non-covalently bound to the microarray surface. Each reaction site was then incubated with 30 μl of undiluted participant plasma for two hours at room temperature in a humidified chamber to enable reactions between IgE and allergen components. Following a washing step, each microarray was incubated for 30 minutes with 30 μl of fluorescence-labeled anti-human IgE detection antibody. Unbound detection antibody was washed off and the fluorescence intensity of each microarray measured using a scanner (LuxScan 10K/A, CapitalBio, Beijing, China). Analysis of the resultant digitalized images was done using Phadia Microarray Image Analysis software (Thermo Fisher Scientific). Results were reported in arbitrary semi-quantitative ISAC Standardised Units (ISU).

## Microarray detection of N-glycan-specific IgE

Each reaction site on each microarray slide included fluorescently-labeled bovine serum albumin (BSA) as a printing control. The NEXTERION®-coated microarray slides [Schott AG, Germany] used in the IgE binding assay were blocked (during microarray construction) with 50mM ethanolamine in 50mM sodium borate buffer pH 9.0, and stored at -20°C. On the day of the binding assay, they were thawed at room temperature (RT) and covered with silicone gaskets to create seven wells with printed microarrays per slide. Each microarray was incubated with 300 μl of a 1:30 plasma dilution in 1% BSA - 0.01% Tween20 for one hour at RT while shaking. After sequential washes with PBS-0.05% Tween20 and PBS, the slides were incubated for 30 minutes at RT in the dark with PromoFluor 647 (VWR, USA)-labeled anti-human IgE clone MH25-1 (Sanquin, Amsterdam, Netherlands) [diluted 1/150 in PBS-0.01% Tween20], while shaking. After a final wash with PBS-0.05% Tween20, PBS and deionised water, sequentially, the slides were dried and kept in the dark until scanning. The slides were scanned for fluorescence at a 10μm resolution with a G2565BA scanner (Agilent Technologies, CA, USA) using a 633nm laser.

## Glycan microarray image processing

Using GenePix Pro 7.0 software (Molecular Devices, CA, USA), a spot-finding algorithm was used to align and re-size fluorescence spots in the microarray images, without setting a composite pixel intensity threshold. Data on median fluorescence intensity (MFI) for each spot and the local background were then exported to Microsoft Excel software. In all analyses, MFIs that were highlighted as artefacts by the GenePix Pro 7.0 software were excluded. Further processing of IgE MFIs in Excel was done as described by Amoah *et al.,*^3^ as follows:for each IgE spot, the ratio of the MFI of the spot to the local background MFI was obtained and then multiplied by the average of background MFI for all the spots on the array. For each of the structures, the average over four spots (or less, in case of unreliable data that were excluded) was then log_2_-transformed.

**Figure S1.** **Koome** **islands and the Entebbe** **peninsula.** *Circular features on Koome islands denote fishing villages. A three-year outcome survey was conducted in these villages as part of the Lake Victoria Island Intervention Study on Worms and Allergy-related diseases [LaVIISWA; ISRCTN47196031], a cluster-randomised trial of community-wide standard versus intensive anthelminthic treatment. White circles represent villages that received standard treatment, red circles represent villages that received intensive treatment and the yellow circle represents the village where the LaVIISWA pilot study was conducted. White dotted demarcation on the Entebbe peninsula denotes the boundary of the municipality.*


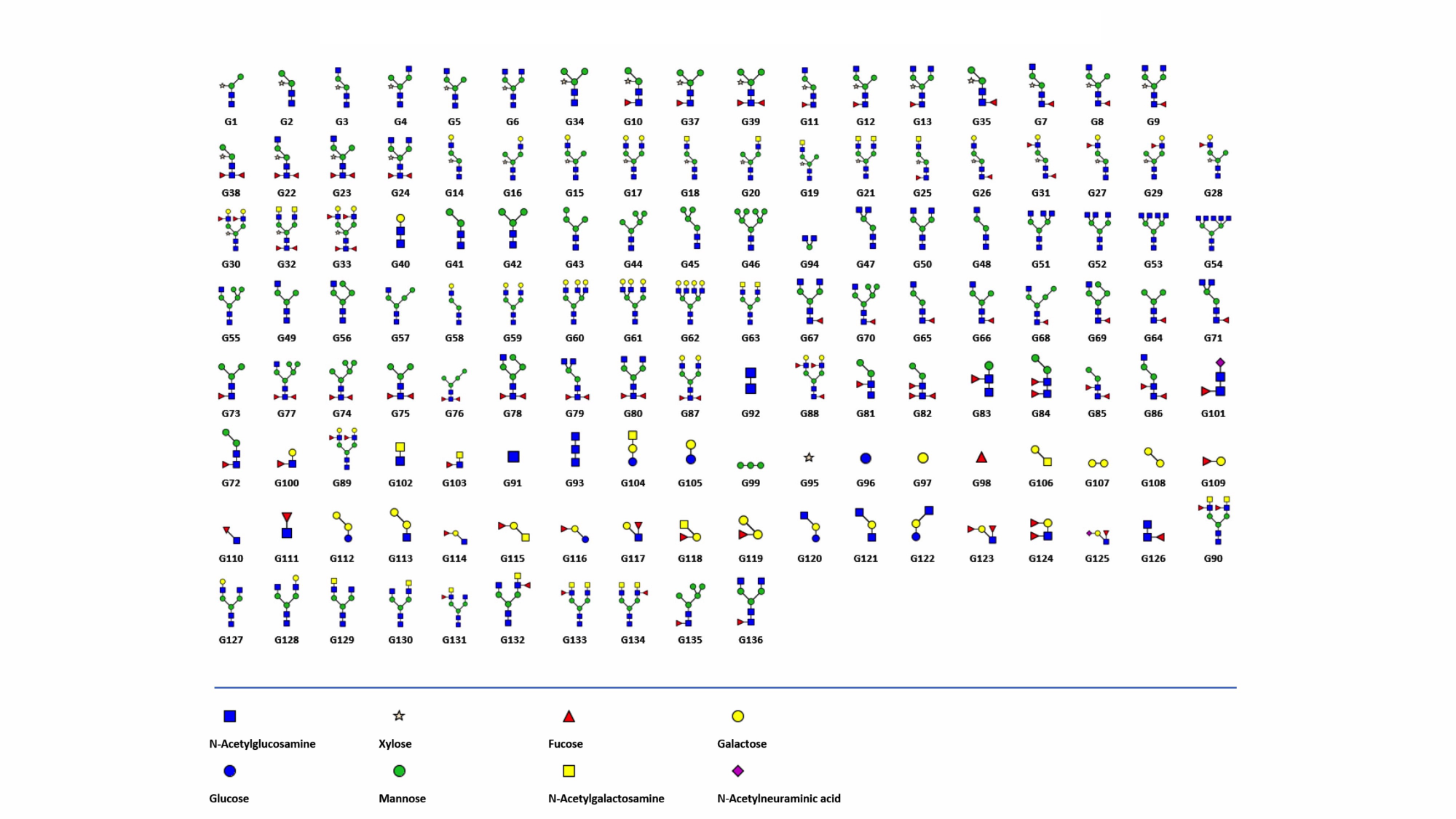


**Figure S2. Collection of synthetic structural variants on the glycan array**

| 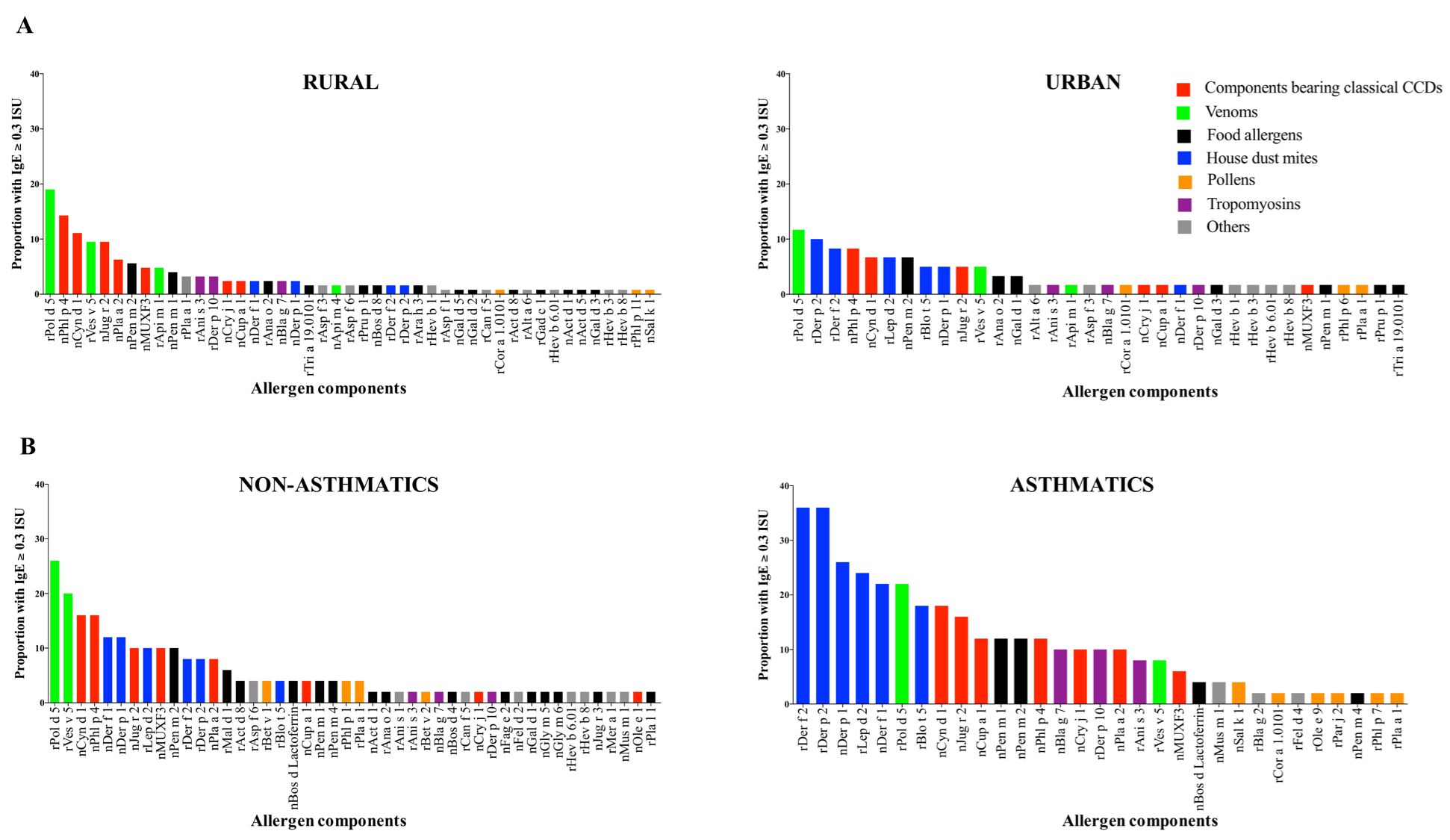 |
| --- |
| **Figure S3. Prevalence of sensitisation (IgE≥0.3 ISU) to allergen components on the ISAC microarray**. *Figure shows only allergen components to which IgE ≥ 0.3 ISU was raised. Natural and recombinant allergens are denoted by the prefixes (****n****) and (****r****), respectively, on the allergen name.*  *ISAC: Immuno Solid-phase Allergen Chip; ISU: ISAC standardised units; CCD: Cross-reactive carbohydrate determinant* |
| 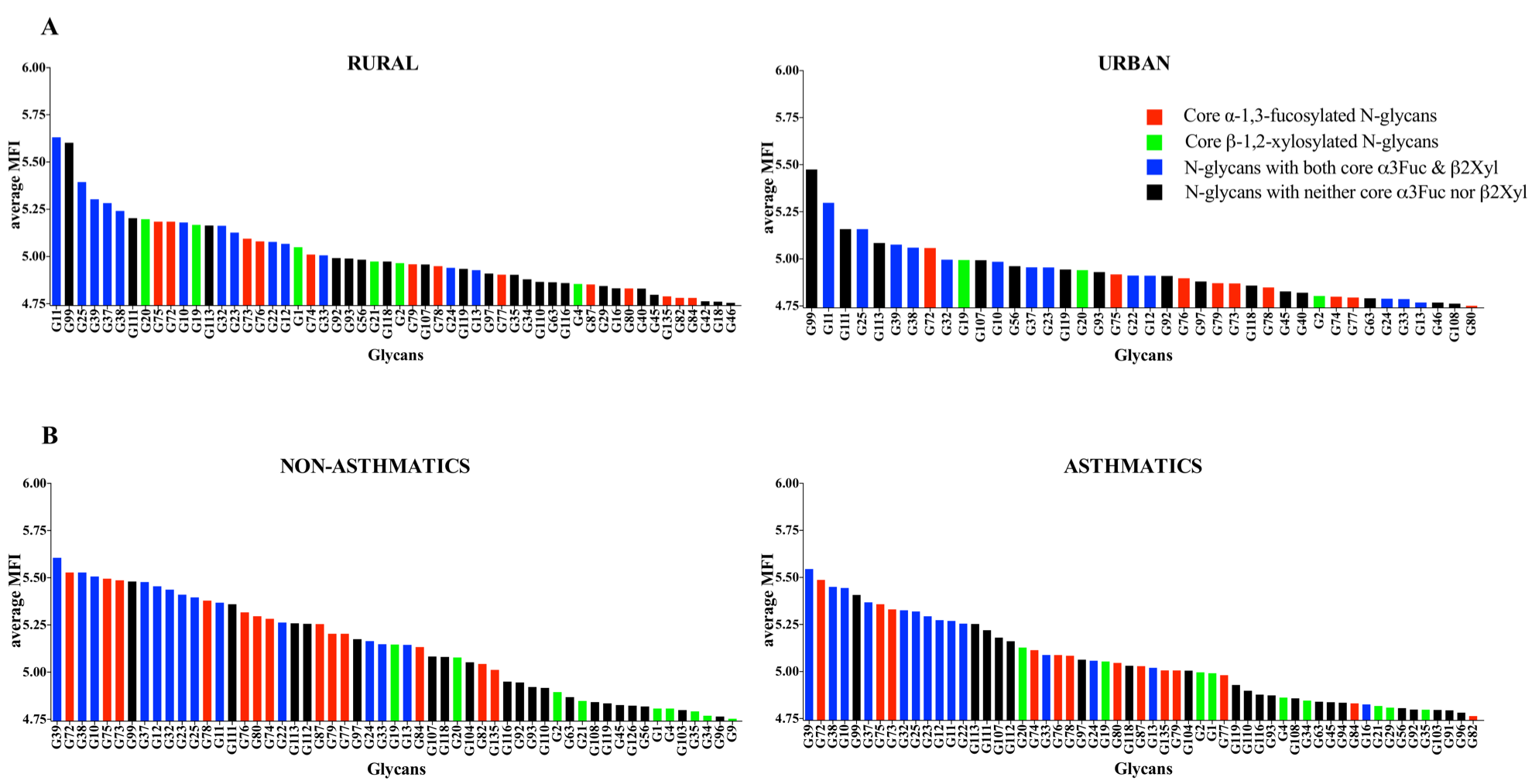 |
| **Figure S4. Responses to structures on the glycan microarray.** *Plasma from rural and the urban survey participants (****panel*** ***A****) and asthmatic and non-asthmatic schoolchildren (****panel*** ***B****) were assessed for IgE reactivity to glycan structural variants with and without α-1,3-fucosylation and β-1,2-xylosylation, on a microarray platform.*  *Graphs show background-subtracted and log_2_-transformed median fluorescence intensities (MFI). Only structures with an average MFI above 4.75 are shown. The entire collection of structures on the microarray is shown in supplementary Figure S2.* |

**Figure S5. ISAC microarray-determined IgE reactivity to venom components.** *Natural and recombinant allergens are denoted by the prefixes (****n****) and (****r****), respectively, on the allergen name. Figure shows data points from individuals with detectable IgE levels. Cut-offs of 0.30 ISU are shown to define the level of ISAC-determined IgE responses that is important for clinical diagnosis of allergic sensitisation.*

*The Mann-Whitney U test was conducted within the framework of a Monte Carlo simulation algorithm based on 1000 permutations (in order to adjust for multiple testing), to assess differences between rural and urban individuals, and between asthmatics and their controls: *p<0.05.*

*ISU: ISAC standardised units*

**Table S1.** Impact of community-based intensive versus standard anthelminthic treatment on IgE profiles in the rural survey

|  |  | n/N (%) / geometric mean | |  | Unadjusted | |  | Adjusted for age and sex | |
| --- | --- | --- | --- | --- | --- | --- | --- | --- | --- |
| **Outcome** | | **Standard** | **Intensive** |  | **RR**^§^ **/ GMR**^#^  **(95% CI)** | **p-value** |  | **RR**^§^ **/ GMR**^#^  **(95% CI)** | **p-value** |
|  |  |  |  |  |  |  |  |  |  |
| **ImmunoCAP-determined IgE** | |  |  |  |  |  |  |  |  |
|  |  |  |  |  |  |  |  |  |  |
|  | *D. pteronyssinus ,* cockroach or peanut positivity (IgE≥0.35kU/L) | 223/390 (57.2%) | 214/390 (54.9%) |  | 0.95 (0.81, 1.10) | 0.46 |  | 0.94 (0.80, 1.10) | 0.41 |
|  | *D. pteronyssinus* positivity (IgE>0.35kU/L) | 134/390 (34.4%) | 130/390 (33.3%) |  | 0.95 (0.76, 1.20) | 0.67 |  | 0.96 (0.77, 1.19) | 0.68 |
|  | German cockroach positivity (IgE>0.35kU/L) | 201/390 (51.5%) | 192/390 (49.2%) |  | 0.94 (0.80, 1.11) | 0.47 |  | 0.94 (0.79, 1.11) | 0.42 |
|  | Peanut positivity (IgE>0.35kU/L) | 59/390 (15.1%) | 55/390 (14.1%) |  | 0.92 (0.59, 1.41) | 0.68 |  | 0.91 (0.58, 1.41) | 0.66 |
|  | Concentration of IgE to *D. pteronyssinus* | GM: 0.158 | GM: 0.129 |  | 0.78 (0.51, 1.17) | 0.22 |  | 0.76 (0.51, 1.13) | 0.17 |
|  | Concentration of IgE to cockroach | GM: 0.342 | GM: 0.289 |  | 0.82 (0.55, 1.22) | 0.31 |  | 0.81 (0.55, 1.20) | 0.28 |
|  | Concentration of IgE to peanut | GM: 0.074 | GM: 0.066 |  | 0.89 (0.64, 1.23) | 0.47 |  | 0.89 (0.65, 1.23) | 0.49 |
|  | |  |  |  |  |  |  |  |  |
| **ISAC-determined IgE sensitisation (IgE≥0.3 ISU)** | |  |  |  |  |  |  |  |  |
|  |  |  |  |  |  |  |  |  |  |
|  | CCD-bearing components | 9/72 (12.5%) | 12/54 (22.2%) |  | 1.87 (0.79, 4.41) | 0.13 |  | 2.46 (0.92, 6.57) | 0.12 |
|  | Venoms | 11/72 (15.3%) | 16/54 (29.6%) |  | 1.87 (0.90, 3.90) | 0.10 |  | 1.23 (0.55, 2.79) | 0.59 |
|  | Dust mite components | 1/72 (1.4%) | 3/54 (5.7%) |  | 2.80 (0.23, 33.85) | 0.41 |  | 3.30 (0.27, 40.80) | 0.36 |
|  |  |  |  |  |  |  |  |  |  |
| **Glycan-specific IgE mean fluorescence intensity** | |  |  |  |  |  |  |  |  |
|  |  |  |  |  |  |  |  |  |  |
|  | 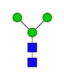 G42 | GM: 4.778 | GM: 4.760 |  | 0.99 (0.99, 1.00) | 0.34 |  | 0.99 (0.99, 1.00) | 0.24 |
|  | 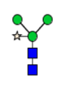 G34 | GM: 4.847 | GM: 4.834 |  | 1.02 (0.98, 1.06) | 0.44 |  | 1.02 (0.98, 1.06) | 0.31 |
|  | 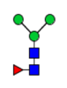 G73 | GM: 5.070 | GM: 5.106 |  | 1.01 (0.96, 1.06) | 0.79 |  | 1.01 (0.96, 1.07) | 0.61 |
|  | 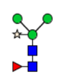 G37 | GM: 5.205 | GM: 5.322 |  | 1.02 (0.96, 1.09) | 0.50 |  | 1.03 (0.97, 1.10) | 0.34 |
|  |  |  |  |  |  |  |  |  |  |
| ***^§^****Risk ratios (mean of the cluster proportions in intensive arm divided by the mean of the cluster proportions in standard arm) were calculated to assess the impact of trial intervention on ImmunoCAP-determined IgE sensitisation to crude house dust mite (D. pteronyssinus), German cockroach and peanut, and on ISAC-determined sensitisation to at least one allergen component within the larger allergen groupings of CCDs, venoms, and dust mite components.*  ***^#^****Geometric mean ratios were calculated to assess the impact of trial intervention on the concentration of ImmunoCAP-determined IgE sensitisation to individual allergens and on IgE mean fluorescence intensities to representative glycans on the array.*  ***RR****: risk ratio;* ***GM:*** *geometric mean;* ***GMR:*** *geometric mean ratio;* ***CI:*** *confidence interval;* ***ISAC****: Immuno Solid-phase Allergen Chip;* ***ISU****: ISAC standardised units* | | | | | | | | | |

**Table S2**. Differences in ISAC-determined IgE reactivity in the rural versus urban setting and among asthmatics versus non-asthmatics

|  |  | **% with detectable IgE, ISAC** | | | **% with IgE≥0.30 ISU, ISAC** | | |  | **% with detectable IgE, ISAC** | | | **% with IgE≥0.30 ISU, ISAC** | | |
| --- | --- | --- | --- | --- | --- | --- | --- | --- | --- | --- | --- | --- | --- | --- |
| Component |  | Rural (n=126) | Urban (n=60) | p | Rural (n=126) | Urban (n=60) | p |  | Non-asthmatics (n=50) | Asthmatics (n=50) | p | Non-asthmatics (n=50) | Asthmatics (n=50) | p |
| **CCD-bearing components** |  |  |  |  |  |  |  |  |  |  |  |  |  |  |
| *nJug r 2* |  | 23.8 | 25.0 | 0.859 | 9.5 | 5.0 | 0.225 |  | 36.0 | 38.0 | 0.836 | 10.0 | 16.0 | 0.375 |
| *nMUXF3* |  | 11.1 | 5.0 | 0.139 | 4.8 | 1.7 | 0.279 |  | 18.0 | 18.0 | 1.000 | 10.0 | 6.0 | 0.463 |
| *nPhl p 4* |  | **28.6** | 10 | **0.005** | 14.3 | 8.3 | 0.181 |  | 34.0 | 32.0 | 0.832 | 16.0 | 12.0 | 0.566 |
| *nCry j 1* |  | **9.5** | 1.7 | **0.040** | 2.4 | 1.7 | 0.612 |  | 8.0 | 14.0 | 0.340 | 2.0 | 10.0 | 0.094 |
| *nCup a 1* |  | 8.7 | 3.3 | 0.148 | 2.4 | 1.7 | 0.612 |  | 12.0 | 14.0 | 0.767 | 4.0 | 12.0 | 0.142 |
| *nCyn d 1* |  | 19.8 | 10.0 | 0.067 | 11.1 | 6.7 | 0.249 |  | 24.0 | 24.0 | 1.000 | 16.0 | 18.0 | 0.791 |
| *nPla a 2* |  | 13.5 | 6.7 | 0.128 | **6.3** | 0.0 | **0.041** |  | 26.0 | 24.0 | 0.818 | 8.0 | 10.0 | 0.728 |
| **Venoms** |  |  |  |  |  |  |  |  |  |  |  |  |  |  |
| *rPol d 5* |  | **39.7** | 20 | **0.008** | 19.0 | 11.7 | 0.207 |  | 38.0 | 40.0 | 0.838 | 26.0 | 22.0 | 0.641 |
| *rVes v 5* |  | **24.6** | 10 | **0.001** | 9.5 | 3.0 | 0.225 |  | 32.0 | 26.0 | 0.511 | 20.0 | 8.0 | 0.085 |
| *rApi m 1* |  | 5.6 | 5.0 | 0.207 | 4.8 | 1.0 | 0.279 |  | **10.0** | **0.0** | **0.023** | 0.0 | 0.0 |  |
| *nApi m 4* |  | 4.8 | 0.0 | 0.090 | 1.6 | 0.0 | 0.458 |  | 2.0 | 0.0 | 0.317 | 0.0 | 0.0 |  |
| **Cross-reactive protein components** |  |  |  |  |  |  |  |  |  |  |  |  |  |  |
| Tropomyosins |  |  |  |  |  |  |  |  |  |  |  |  |  |  |
| *nBla g 7* |  | 4.0 | 1.7 | 0.369 | 2.4 | 1.7 | 0.612 |  | 8.0 | 12.0 | 0.507 | 2.0 | 10.0 | 0.094 |
| *rDer p 10* |  | 3.9 | 1.7 | 0.369 | 3.2 | 1.7 | 0.481 |  | 6.0 | 12.0 | 0.296 | 2.0 | 10.0 | 0.094 |
| *nPen m1* |  | 4.8 | 5.0 | 0.600 | 4.0 | 1.7 | 0.369 |  | 12.0 | 12.0 | 1.000 | 4.0 | 12.0 | 0.142 |
| *rAni s 3* |  | 5.6 | 3.3 | 0.400 | 3.2 | 1.7 | 0.549 |  | 4.0 | 10.0 | 0.242 | 2.0 | 8.0 | 0.171 |
| *rHev b 8* |  | 0.8 | 1.7 | 0.542 | 0.8 | 1.7 | 0.542 |  | 4.0 | 0.0 | 0.155 | 2.0 | 0.0 | 0.317 |
| Polcalcins |  |  |  |  |  |  |  |  |  |  |  |  |  |  |
| *rBet v 4* |  | 0.0 | 0.0 |  | 0.0 | 0.0 |  |  | 0.0 | 2.0 | 0.500 | 0.0 | 0.0 |  |
| *rPhl p 7* |  | 0.8 | 0.0 | 0.677 | 0.0 | 0.0 |  |  | 2.0 | 2.0 | 0.753 | 0.0 | 2.0 | 0.500 |
| Profilins |  |  |  |  |  |  |  |  |  |  |  |  |  |  |
| *rPhl p 12* |  | 1.6 | 0.0 | 0.458 | 0.0 | 0.0 |  |  | 2.0 | 0.0 | 0.500 | 0.0 | 0.0 |  |
| *rBet v 2* |  | 0.0 | 1.7 | 0.323 | 0.0 | 0.0 |  |  | 2.0 | 0.0 | 0.500 | 2.0 | 0.0 | 0.500 |
| *rMer a 1* |  | 0.8 | 1.7 | 0.542 | 0.0 | 0.0 |  |  | 2.0 | 2.0 | 0.753 | 2.0 | 0.0 | 0.500 |
| **Dust mites** |  |  |  |  |  |  |  |  |  |  |  |  |  |  |
| *nDer p 1* |  | 2.4 | 8.3 | 0.073 | 2.4 | 5.0 | 0.296 |  | 16.0 | 28.0 | 0.149 | 12.0 | 26.0 | 0.075 |
| *rDer p 2* |  | 2.4 | **13.3** | **0.006** | 1.6 | **10.0** | **0.015** |  | 10.0 | **36.0** | **0.002** | 8.0 | **36.0** | **<0.001** |
| *nDer f 1* |  | 6.3 | 5.0 | 0.502 | 2.4 | 1.7 | 0.612 |  | 16.0 | 28.0 | 0.149 | 12.0 | 22.0 | 0.185 |
| *rDer f 2* |  | 2.4 | 8.3 | 0.073 | 1.6 | **8.3** | **0.036** |  | 14.0 | **36.0** | **0.012** | 8.0 | **36.0** | **<0.001** |
| *rBlo t 5* |  | 0.8 | **6.7** | **0.038** | 0.0 | **5.0** | **0.032** |  | 8.0 | 20.0 | 0.085 | 4.0 | **18.0** | **0.026** |
| *rLep d 2* |  | 0.0 | **6.6** | **0.010** | 0.0 | **6.7** | **0.010** |  | 12.0 | **32.0** | **0.016** | 8.0 | **24.0** | **0.029** |
|  |  |  |  |  |  |  |  |  |  |  |  |  |  |  |
| **Cockroach** |  |  |  |  |  |  |  |  |  |  |  |  |  |  |
| *rBla g 1* |  | 0.0 | 0.0 |  | 0.0 | 0.0 |  |  | 0.0 | 0.0 |  | 0.0 | 0.0 |  |
| *rBla g 2* |  | 0.0 | 1.7 | 0.323 | 0.0 | 0.0 |  |  | 2.0 | 10.0 | 0.102 | 0.0 | 2.0 | 0.500 |
| *rBla g 5* |  | 0.8 | 0.0 | 0.677 | 0.0 | 0.0 |  |  | 0.0 | 2.0 | 0.500 | 0.0 | 0.0 |  |
| **Food allergens** |  |  |  |  |  |  |  |  |  |  |  |  |  |  |
| *rAna o 2* |  | 4.8 | 6.7 | 0.411 | 2.4 | 3.3 | 0.519 |  | 4.0 | 4.0 | 0.691 | 2.0 | 0.0 | 0.500 |
| *rApi g 1* |  | 2.4 | **10.0** | **0.033** | 0.0 | 0.0 |  |  | 0.0 | 0.0 |  | 0.0 | 0.0 |  |
| *nPen m2* |  | 4.8 | 5.0 | 0.600 | 5.6 | 6.7 | 0.498 |  | 16.0 | 16.0 | 1.000 | 10.0 | 12.0 | 0.500 |
| *nPen m 4* |  | 0.8 | 0.0 | 0.677 | 0.0 | 0.0 |  |  | 6.0 | 2.0 | 0.309 | 4.0 | 2.0 | 0.500 |
| *nBos d 4* |  | 0.8 | 0.0 | 0.677 | 0.0 | 0.0 |  |  | 2.0 | 4.0 | 0.500 | 2.0 | 0.0 | 0.500 |
| *nBos d 6* |  | 0.0 | 0.0 |  | 0.0 | 0.0 |  |  | 10.0 | 6.0 | 0.357 | 0.0 | 0.0 |  |
| *nBos d 8* |  | 3.2 | 0.0 | 0.207 | 1.6 | 0.0 | 0.458 |  | 0.0 | 0.0 |  | 0.0 | 0.0 |  |
| *nBos d lactoferrin* |  | 2.4 | 0.0 | 0.308 | 0.0 | 0.0 |  |  | 8.0 | 12.0 | 0.370 | 4.0 | 4.0 | 0.691 |
| *nGal d 1* |  | 3.9 | 8.3 | 0.186 | 0.0 | 3.3 | 0.103 |  | 2.0 | 0.0 | 0.500 | 2.0 | 0.0 | 0.500 |
| *nGal d 2* |  | 3.2 | 3.3 | 0.631 | 0.8 | 0.0 | 0.677 |  | 0.0 | 0.0 |  | 0.0 | 0.0 |  |
| *nGal d 3* |  | 0.8 | 3.3 | 0.244 | 0.8 | 1.7 | 0.542 |  | 2.0 | 0.0 | 0.500 | 0.0 | 0.0 |  |
| *nGal d 5* |  | 3.9 | 0.0 | 0.139 | 0.8 | 0.0 | 0.677 |  | 0.0 | 2.0 | 0.500 | 0.0 | 0.0 |  |
| *rGly m 4* |  | 0.0 | 0.0 |  | 0.0 | 0.0 |  |  | 0.0 | 0.0 |  | 0.0 | 0.0 |  |
| *nGly m 5* |  | 0.0 | 0.0 |  | 0.0 | 0.0 |  |  | 2.0 | 0.0 | 0.500 | 2.0 | 0.0 | 0.500 |
| *nGly m 6* |  | 0.0 | 1.7 | 0.323 | 0.0 | 0.0 |  |  | 6.0 | 6.0 | 0.661 | 2.0 | 0.0 | 0.500 |
| *rMal d 1* |  | 0.8 | **13.3** | **0.001** | 0.0 | 0.0 |  |  | 10.0 | 4.0 | 0.218 | 6.0 | 0.0 | 0.121 |
| *rPru p 1* |  | 3.9 | 3.3 | 0.596 | 1.6 | 1.7 | 0.692 |  | 6.0 | 4.0 | 0.500 | 0.0 | 0.0 |  |
| *rPru p 3* |  | 0.0 | 0.0 |  | 0.0 | 0.0 |  |  | 0.0 | 2.0 | 0.500 | 0.0 | 0.0 |  |
| *rTri a 14* |  | 2.4 | 0.0 | 0.308 | 0.0 | 0.0 |  |  | 0.0 | 0.0 |  | 0.0 | 0.0 |  |
| *rTri a 19.0101* |  | 7.1 | 5.0 | 0.420 | 1.6 | 1.7 | 0.692 |  | 0.0 | 0.0 |  | 0.0 | 0.0 |  |
| *nTri a aA_TI* |  | 0.8 | 0.0 | 0.677 | 0.0 | 0.0 |  |  | 2.0 | 4.0 | 0.500 | 0.0 | 0.0 |  |
| *nAct d 1* |  | 0.8 | 0.0 | 0.677 | 0.8 | 0.0 | 0.677 |  | 6.0 | 0.0 | 0.121 | 2.0 | 0.0 | 0.500 |
| *nAct d 2* |  | 0.8 | 0.0 | 0.677 | 0.0 | 0.0 |  |  | 0.0 | 0.0 |  | 0.0 | 0.0 |  |
| *nAct d 5* |  | 0.8 | 0.0 | 0.677 | 0.8 | 0.0 | 0.677 |  | 0.0 | 0.0 |  | 0.0 | 0.0 |  |
| *rAct d 8* |  | 1.6 | 1.6 | 0.692 | 0.8 | 0.0 | 0.677 |  | 8.0 | 4.0 | 0.339 | 4.0 | 0.0 | 0.247 |
| *rAra h 1* |  | 0.8 | 0.0 | 0.677 | 0.0 | 0.0 |  |  | 0.0 | 4.0 | 0.247 | 0.0 | 0.0 |  |
| *rAra h 2* |  | 0.8 | 0.0 | 0.677 | 0.0 | 0.0 |  |  | 0.0 | 0.0 |  | 0.0 | 0.0 |  |
| *rAra h 3* |  | 1.6 | 0.0 | 0.458 | 1.6 | 0.0 | 0.458 |  | 0.0 | 0.0 |  | 0.0 | 0.0 |  |
| *nAra h 6* |  | 0.0 | 0.0 |  | 0.0 | 0.0 |  |  | 0.0 | 0.0 |  | 0.0 | 0.0 |  |
| *rAra h 8* |  | 0.0 | 3.3 | 0.103 | 0.0 | 0.0 |  |  | 0.0 | 6.0 | 0.121 | 0.0 | 0.0 |  |
| *rAra h 9* |  | 0.8 | 0.0 | 0.677 | 0.0 | 0.0 |  |  | 0.0 | 2.0 | 0.500 | 0.0 | 0.0 |  |
| *rBer e 1* |  | 0.0 | 0.0 |  | 0.0 | 0.0 |  |  | 0.0 | 0.0 |  | 0.0 | 0.0 |  |
| *rCor a 1.0401* |  | 0.0 | 1.7 | 0.323 | 0.0 | 0.0 |  |  | 2.0 | 0.0 | 0.500 | 0.0 | 0.0 |  |
| *rCor a 8* |  | 0.0 | 0.0 |  | 0.0 | 0.0 |  |  | 0.0 | 0.0 |  | 0.0 | 0.0 |  |
| *nCor a 9* |  | 0.0 | 0.0 |  | 0.0 | 0.0 |  |  | 0.0 | 0.0 |  | 0.0 | 0.0 |  |
| *nJug r 1* |  | 0.0 | 0.0 |  | 0.0 | 0.0 |  |  | 0.0 | 2.0 | 0.500 | 0.0 | 0.0 |  |
| *nJug r 3* |  | 0.0 | 0.0 |  | 0.0 | 0.0 |  |  | 2.0 | 0.0 | 0.500 | 2.0 | 0.0 | 0.500 |
| *rPla l 1* |  | 0.0 | 0.0 |  | 0.0 | 0.0 |  |  | 6.0 | 0.0 | 0.121 | 2.0 | 0.0 | 0.500 |
| *nSes i 1* |  | 0.0 | 0.0 |  | 0.0 | 0.0 |  |  | 0.0 | 0.0 |  | 0.0 | 0.0 |  |
| *nFag e 2* |  | 0.0 | 0.0 |  | 0.0 | 0.0 |  |  | 2.0 | 0.0 | 0.500 | 2.0 | 0.0 | 0.500 |
| **Pollen** |  |  |  |  |  |  |  |  |  |  |  |  |  |  |
| *rAln g 1* |  | 0.8 | 0.0 | 0.677 | 0.0 | 0.0 |  |  | 0.0 | 0.0 |  | 0.0 | 0.0 |  |
| *nArt v 1* |  | 0.0 | 0.0 |  | 0.0 | 0.0 |  |  | 0.0 | 0.0 |  | 0.0 | 0.0 |  |
| *nArt v 3* |  | 0.0 | 0.0 |  | 0.0 | 0.0 |  |  | 2.0 | 0.0 | 0.500 | 0.0 | 0.0 |  |
| *rBet v 1* |  | 1.6 | 0.0 | 0.458 | 0.0 | 0.0 |  |  | 10.0 | 8.0 | 0.500 | 4.0 | 0.0 | 0.247 |
| *rChe a 1* |  | 0.8 | 0.0 | 0.677 | 0.0 | 0.0 |  |  | 2.0 | 4.0 | 0.500 | 0.0 | 0.0 |  |
| *rMer a 1* |  | 0.8 | 1.7 | 0.542 | 0.0 | 0.0 |  |  | 2.0 | 2.0 | 0.753 | 2.0 | 0.0 | 0.500 |
| *nOle e 7* |  | 0.0 | 0.0 |  | 0.0 | 0.0 |  |  | 0.0 | 0.0 |  | 0.0 | 0.0 |  |
| *rOle e 9* |  | 2.4 | 3.3 | 0.519 | 0.0 | 0.0 |  |  | 0.0 | 4.0 | 0.247 | 0.0 | 2.0 | 0.500 |
| *rPar j 2* |  | 0.0 | 0.0 |  | 0.0 | 0.0 |  |  | 2.0 | 4.0 | 0.500 | 0.0 | 2.0 | 0.500 |
| *rCor a 1.0101* |  | 2.4 | 3.3 | 0.519 | 0.8 | 1.7 | 0.542 |  | 4.0 | 6.0 | 0.500 | 0.0 | 2.0 | 0.500 |
| *rPhl p 1* |  | 0.0 | 1.7 | 0.323 | 0.0 | 0.0 |  |  | 6.0 | 2.0 | 0.309 | 4.0 | 0.0 | 0.247 |
| *rPhl p 11* |  | 0.8 | 1.7 | 0.542 | 0.8 | 0.0 | 0.677 |  | 0.0 | 4.0 | 0.247 | 0.0 | 0.0 |  |
| *rPhl p 12* |  | 1.6 | 0.0 | 0.458 | 0.0 | 0.0 |  |  | 2.0 | 0.0 | 0.500 | 0.0 | 0.0 |  |
| *rPhl p 2* |  | 0.0 | 0.0 |  | 0.0 | 0.0 |  |  | 0.0 | 0.0 |  | 0.0 | 0.0 |  |
| *rPhl p 5* |  | 0.8 | 0.0 | 0.677 | 0.0 | 0.0 |  |  | 4.0 | 0.0 | 0.247 | 0.0 | 0.0 |  |
| *rPhl p 6* |  | 1.6 | 1.7 | 0.692 | 0.0 | 1.7 | 0.323 |  | 0.0 | 0.0 |  | 0.0 | 0.0 |  |
| *rPhl p 7* |  | 0.8 | 0.0 | 0.677 | 0.0 | 0.0 |  |  | 2.0 | 2.0 | 0.753 | 0.0 | 2.0 | 0.500 |
| *rPla a 1* |  | 6.4 | 3.3 | 0.319 | 3.2 | 1.7 | 0.481 |  | 10.0 | 6.0 | 0.357 | 4.0 | 2.0 | 0.500 |
| *rPla a 3* |  | 0.0 | 0.0 |  | 0.0 | 0.0 |  |  | 0.0 | 0.0 |  | 0.0 | 0.0 |  |
| *nSal k 1* |  | 0.8 | 0.0 | 0.677 | 0.8 | 0.0 | 0.677 |  | 4.0 | 10.0 | 0.218 | 0.0 | 4.0 | 0.247 |
| **Fungi** |  |  |  |  |  |  |  |  |  |  |  |  |  |  |
| *rAlt a 1* |  | 0.0 | 0.0 |  | 0.0 | 0.0 |  |  | 0.0 | 0.0 |  | 0.0 | 0.0 |  |
| *rAlt a 6* |  | 1.6 | 3.3 | 0.388 | 0.8 | 1.7 | 0.542 |  | 4.0 | 4.0 | 0.691 | 0.0 | 0.0 |  |
| *nAmb a 1* |  | 0.8 | 0.0 | 0.677 | 0.0 | 0.0 |  |  | 0.0 | 0.0 |  | 0.0 | 0.0 |  |
| *rAsp f 1* |  | 5.6 | 8.3 | 0.334 | 0.8 | 0.0 | 0.677 |  | 4.0 | 0.0 | 0.247 | 0.0 | 0.0 |  |
| *rAsp f 3* |  | 6.4 | 11.7 | 0.169 | 1.6 | 1.7 | 0.692 |  | 2.0 | 2.0 | 0.753 | 0.0 | 0.0 |  |
| *rAsp f 6* |  | 3.9 | 1.7 | 0.369 | 1.6 | 0.0 | 0.458 |  | 4.0 | 0.0 | 0.247 | 4.0 | 0.0 | 0.247 |
| *rCla h 8* |  | 0.8 | 0.0 | 0.677 | 0.0 | 0.0 |  |  | 0.0 | 2.0 | 0.500 | 0.0 | 0.0 |  |
| **Other, animal** |  |  |  |  |  |  |  |  |  |  |  |  |  |  |
| *rAni s 1* |  | 0.0 | 0.0 |  | 0.0 | 0.0 |  |  | 2.0 | 0.0 | 0.500 | 2.0 | 0.0 | 0.500 |
| *rCan f 2* |  | 0.8 | 1.7 | 0.542 | 0.0 | 0.0 |  |  | 0.0 | 0.0 |  | 0.0 | 0.0 |  |
| *nCan f 3* |  | 0.0 | 0.0 |  | 0.0 | 0.0 |  |  | 0.0 | 4.0 | 0.247 | 0.0 | 0.0 |  |
| *rCan f 5* |  | 2.4 | 1.7 | 0.612 | 0.8 | 0.0 | 0.677 |  | 4.0 | 0.0 | 0.247 | 2.0 | 0.0 | 0.500 |
| *rEqu c 1* |  | 0.8 | 0.0 | 0.677 | 0.0 | 0.0 |  |  | 0.0 | 0.0 |  | 0.0 | 0.0 |  |
| *nEqu c 3* |  | 0.0 | 0.0 |  | 0.0 | 0.0 |  |  | 0.0 | 2.0 | 0.500 | 0.0 | 0.0 |  |
| *rFel d 1* |  | 0.0 | 0.0 |  | 0.0 | 0.0 |  |  | 0.0 | 0.0 |  | 0.0 | 0.0 |  |
| *nFel d 2* |  | 2.4 | 6.7 | 0.153 | 0.0 | 0.0 |  |  | 6.0 | 10.0 | 0.357 | 2.0 | 0.0 | 0.500 |
| *rFel d 4* |  | **0.8** | **6.7** | **0.038** | 0.0 | 0.0 |  |  | 2.0 | 2.0 | 0.753 | 0.0 | 2.0 | 0.500 |
| *nMus m 1* |  | 0.0 | 0.0 |  | 0.0 | 0.0 |  |  | 2.0 | 6.0 | 0.309 | 2.0 | 4.0 | 0.500 |
| **Latex** |  |  |  |  |  |  |  |  |  |  |  |  |  |  |
| *rHev b 1* |  | 1.6 | 1.7 | 0.692 | 1.6 | 1.7 | 0.692 |  | 2.0 | 0.0 | 0.500 | 0.0 | 0.0 |  |
| *rHev b 3* |  | 0.8 | 1.7 | 0.542 | 0.8 | 1.7 | 0.542 |  | 0.0 | 0.0 |  | 0.0 | 0.0 |  |
| *rHev b 5* |  | 0.8 | 0.0 | 0.677 | 0.0 | 0.0 |  |  | 0.0 | 0.0 |  | 0.0 | 0.0 |  |
| *rHev b 6.01* |  | 1.6 | 1.7 | 0.692 | 0.8 | 1.7 | 0.542 |  | 2.0 | 0.0 | 0.500 | 2.0 | 0.0 | 0.500 |
| *rHev b 8* |  | 0.8 | 1.7 | 0.542 | 0.8 | 1.7 | 0.542 |  | 4.0 | 0.0 | 0.247 | 2.0 | 0.0 | 0.500 |
|  | | | | | | | | | | | | | | |
| *Table shows proportions of participants that had an ISAC-detectable IgE response or were sensitised (IgE≥0.3 ISU) to components on the array. The Chi-square / Fisher’s exact test was conducted to assess differences in proportions between rural and urban individuals, and between asthmatics and their controls. These tests were conducted within the framework of a Monte Carlo simulation algorithm based on 1000 permutations in order to adjust for multiple testing. Percentages that were significantly higher in one group compared to the other (p≤0.05) are highlighted in bold.*  ***ISAC****: Immuno Solid-phase Allergen Chip;* ***ISU****: ISAC standardised units* | | | | | | | | | | | | | | |

**Table S3.** ISAC-determined IgE reactivity among helminth infected and uninfected rural survey participants

| **Allergen group** |  | **% with detectable IgE, ISAC array** | | |  | **% with IgE≥0.30 ISU, ISAC array** | | |
| --- | --- | --- | --- | --- | --- | --- | --- | --- |
|  |  | **Any worm infection (n=62)** | **Uninfected (n=50)** | p |  | **Any worm infection (n=62)** | **Uninfected (n=50)** | p |
| CCD-bearing components |  | 38.7 | 26.0 | 0.155 |  | 17.7 | 14.0 | 0.592 |
| Venoms |  | **59.7** | **32.0** | **0.004** |  | **30.7** | **12.0** | **0.018** |
| Dust mites |  | 8.1 | 6.0 | 0.484 |  | 3.2 | 4.0 | 0.606 |
| Cockroach |  | 4.8 | 2.0 | 0.394 |  | 3.2 | 2.0 | 0.581 |
| Peanuts |  | 1.6 | 2.0 | 0.696 |  | 1.6 | 2.0 | 0.696 |
| Food components (including peanuts) |  | 51.6 | 38.0 | 0.150 |  | 25.8 | 18.0 | 0.324 |
| Pollen |  | 38.7 | 30.0 | 0.336 |  | 16.1 | 16.0 | 0.985 |
| Fungal allergens |  | **19.4** | **2.0** | **0.003** |  | 6.5 | 0.0 | 0.090 |
| Domesticated animals |  | 4.8 | 4.0 | 0.601 |  | 1.6 | 0.0 | 0.554 |
|  |  |  |  |  |  |  |  |  |
|  |  | ***Sm+* (n=53)*** | ***Sm-* (n=58)** |  |  | ***Sm+* (n=37)** | ***Sm-* (n=75)** |  |
| CCD-bearing components |  | 35.9 | 29.3 | 0.462 |  | 15.1 | 15.5 | 0.951 |
| Venoms |  | **60.4** | **34.5** | **0.006** |  | **30.2** | **13.8** | **0.036** |
| Dust mites |  | 9.4 | 5.2 | 0.309 |  | 3.8 | 3.5 | 0.656 |
| Cockroach |  | 3.8 | 3.5 | 0.656 |  | 1.9 | 3.5 | 0.534 |
| Peanuts |  | 0.0 | 1.7 | 0.523 |  | 0.0 | 1.7 | 0.523 |
| Food components (including peanuts) |  | 49.1 | 41.4 | 0.417 |  | 20.8 | 22.4 | 0.832 |
| Pollen |  | 35.9 | 32.8 | 0.732 |  | 15.1 | 15.5 | 0.951 |
| Fungal allergens |  | **18.9** | **3.5** | **0.009** |  | 5.7 | 1.7 | 0.276 |
| Domesticated animals |  | 5.7 | 3.5 | 0.457 |  | 1.9 | 0.0 | 0.477 |
|  |  |  |  |  |  |  |  |  |
|  |  | **Any nematode infection^¶^ (n=26)** | **Uninfected (n=86)** |  |  | **Any nematode infection^¶^ (n=26)** | **Uninfected (n=86)** |  |
| CCD-bearing components |  | 38.5 | 31.4 | 0.502 |  | 19.2 | 15.1 | 0.617 |
| Venoms |  | 57.7 | 44.2 | 0.227 |  | 23.1 | 22.1 | 0.916 |
| Dust mites |  | 7.7 | 6.9 | 0.595 |  | 0.0 | 4.7 | 0.342 |
| Cockroach |  | 3.9 | 3.5 | 0.658 |  | 3.9 | 2.3 | 0.551 |
| Peanuts |  | 3.9 | 1.2 | 0.412 |  | 3.9 | 1.2 | 0.412 |
| Food components (including peanuts) |  | 53.9 | 43.0 | 0.332 |  | 34.6 | 18.6 | 0.086 |
| Pollen |  | 38.5 | 33.7 | 0.657 |  | 15.4 | 16.3 | 0.592 |
| Fungal allergens |  | **23.1** | **8.1** | **0.037** |  | **11.5** | **1.2** | **0.038** |
| Domesticated animals |  | 0.0 | 5.8 | 0.260 |  | 0.0 | 1.2 | 0.768 |
| *Table shows proportions of rural participants (categorised by helminth infection status) that had an ISAC-detectable IgE response or were sensitised (IgE≥0.3 ISU) to at least one allergen component within the larger allergen groupings of CCDs, venoms, dust mites, cockroach, peanut, food, pollen, fungi and domesticated animals. Helminth prevalence was low in the urban survey and in the asthma study, hence associations with helminths were not examined there. Chi-square / Fisher’s exact tests were conducted to assess differences between helminth infected and uninfected participants. These tests were conducted within the framework of a Monte Carlo simulation algorithm based on 1000 permutations in order to adjust for multiple testing. Percentages that were significantly higher in one group compared to the other (p≤0.05) are highlighted in bold.*  **Schistosoma mansoni infection determined by Kato-Katz and/or PCR*  *^¶^Infection with any of Ascaris lumbricoides, Trichuris trichiura (assessed by KK), Necator americanus, Strongyloides stercoralis (assessed by PCR) and Mansonella perstans (assessed by modified Knott’s method).*  ***ISAC****: Immuno Solid-phase Allergen Chip;* ***ISU****: ISAC standardised units* | | | | | | | | |

| 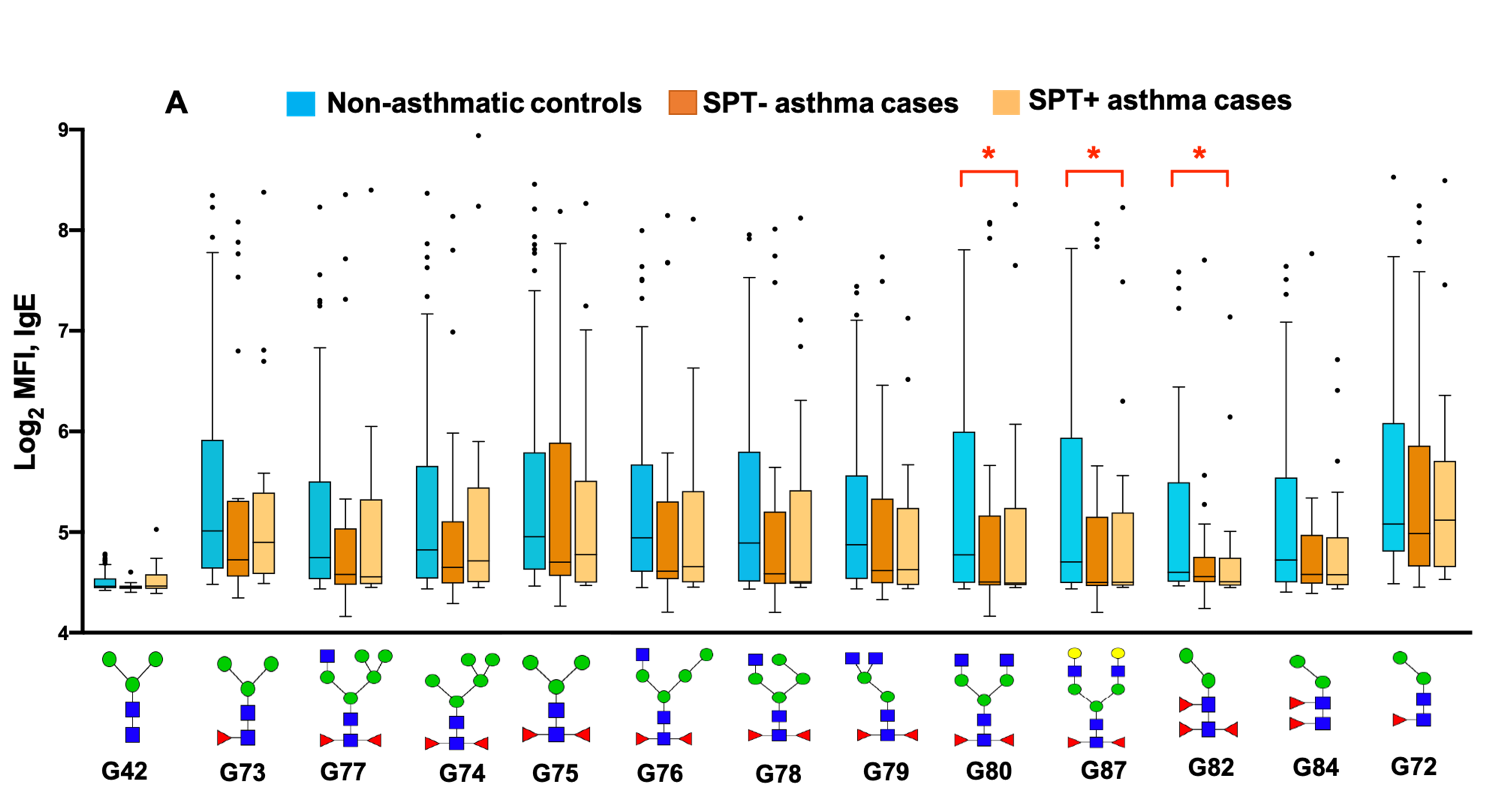 |
| --- |
| 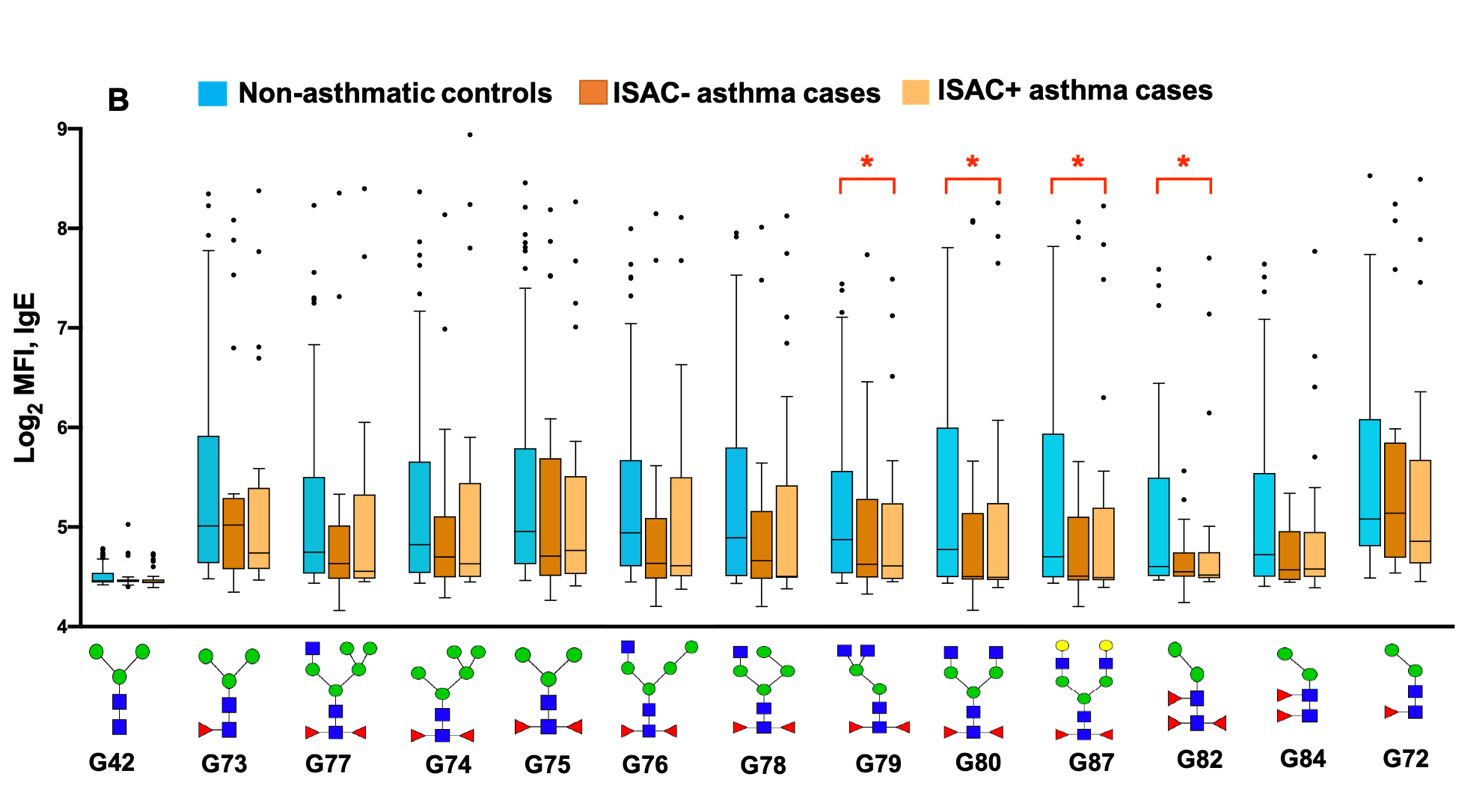 |
| **Figure S6. Associations between ‘asthma phenotype’ and IgE reactivity to core α-1,3-fucosylated N-glycan structures.**  ***A.*** *We created asthma groupings basing on SPT reactivity to at least one allergen extract. The groups were termed “allergic” (or SPT+ asthma cases) and “non-allergic” (or SPT- asthma cases). Box-and-whisker plots show MFIs representing IgE reactivity to core α-1,3-fucosylated N-glycan structures among non-asthmatic controls, SPT negative asthma cases and SPT positive asthma cases.*  ***B.*** *We created asthma groupings basing on sensitisation to at least one recombinant component on the ISAC microarray, which we termed “allergic” (or ISAC+ asthma cases; IgE≥0.3 ISU) and “non-allergic” (or ISAC- asthma cases; IgE<0.3 ISU). Box-and-whisker plots show MFIs representing IgE reactivity to core α-1,3-fucosylated N-glycan structures among non-asthmatic controls, ISAC negative asthma cases and ISAC asthma cases.*  *Horizontal lines in the plots represent medians and boxes denote interquartile ranges (IQR). Whiskers were drawn using the Tukey method (1.5 times IQR). Individual points represent outliers (> 1.5 times IQR away from median).*  *The Mann-Whitney U test was conducted within the framework of a Monte Carlo simulation algorithm based on 1000 permutations (in order to adjust for multiple testing), to assess differences between 1) non-asthmatic schoolchildren and “allergic” asthma cases, 2) non-asthmatic schoolchildren and “non-allergic” asthma cases, 3) “allergic” and “non-allergic” asthma cases.*  **p<0.05 for comparison between non-asthmatic schoolchildren and “allergic” asthma cases. P values were greater than 0.05 for all other comparisons.* |

**References**

1. Nampijja M, Webb EL, Kaweesa J, et al. The Lake Victoria island intervention study on worms and allergy-related diseases (LaVIISWA): study protocol for a randomised controlled trial. *Trials.* 2015;16(1):187.

2. Sanya RE, Nkurunungi G, Hoek Spaans R, et al. The Impact of Intensive Versus Standard Anthelminthic Treatment on Allergy-related Outcomes, Helminth Infection Intensity, and Helminth-related Morbidity in Lake Victoria Fishing Communities, Uganda: Results From the LaVIISWA Cluster-randomized Trial. *Clinical Infectious Diseases.* 2018:ciy761-ciy761.

3. Amoah AS, Asuming-Brempong EK, Obeng BB, et al. Identification of dominant anti-glycan IgE responses in school children by glycan microarray. *The Journal of allergy and clinical immunology.* 2018;141(3):1130-1133.

4. Onell A, Hjalle L, Borres MP. Exploring the temporal development of childhood IgE profiles to allergen components. *Clinical and translational allergy.* 2012;2(1):24.

5. van Diepen A, Smit CH, van Egmond L, et al. Differential anti-glycan antibody responses in Schistosoma mansoni-infected children and adults studied by shotgun glycan microarray. *PLoS Negl Trop Dis.* 2012;6(11):e1922.
